# Mammalian synthetic gene circuits for biopharmaceutical development & manufacture

**DOI:** 10.1038/s41540-025-00621-y

**Published:** 2025-12-02

**Authors:** Sheryl Li Yan Lim, Sofia Gialamoidou, Rajinder Kaur, Ioscani Jimenez del Val

**Affiliations:** School of Chemical & Bioprocess Engineering, University College Dublin D04 V1W8, Dublin, Ireland

**Keywords:** Biological techniques, Biotechnology, Computational biology and bioinformatics, Engineering

## Abstract

This paper reviews the design and application of mammalian synthetic gene circuits for biopharmaceutical manufacturing. It discusses key design principles and outlines transcription factors, DNA-binding proteins, and RNA as input and regulatory modules, while also presenting computational modelling as a driver for circuit optimisation. The review highlights potential applications towards the production of next-generation biotherapeutics by providing examples on monoclonal antibody glycosylation control, CAR-T cell therapy safety, and gene therapy viral vector yields.

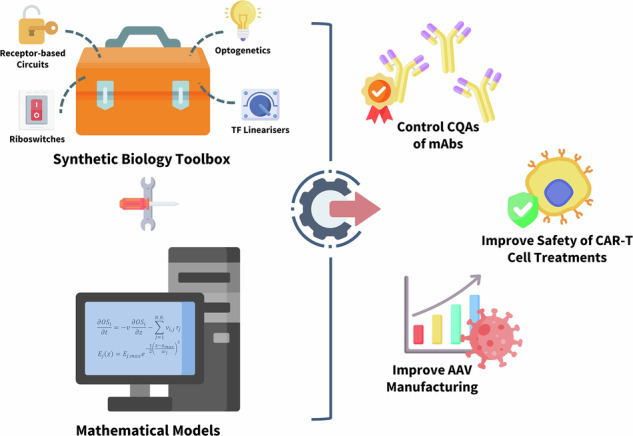

## Introduction

Biopharmaceuticals are projected to exceed a global market capitalisation of U.S. $900 billion by 2030^1^. Despite the COVID-19 pandemic, monoclonal antibodies (mAbs) sales represented the majority of total protein-based therapeutics sales in 2022. In addition, there has been a notable increase in biosimilar approval rates over the past 5 years. However, a large proportion of these biosimilars do not require extensive post-translational modifications (e.g., glycosylation), thus enabling their production in relatively cheaper non-mammalian systems^2^.

The rapidly increasing demand of biologics is driving the need for maintaining product quality while increasing yields. This has led to the development of advanced process analytical technologies (PAT) to track biopharmaceutical production by providing real-time measurements of critical quality attributes (CQAs)^3^. However, closed-loop control systems remain a challenge due to the absence of actuators which can modulate—at the biological level—CQAs as a function of PAT signals. The synthetic biology toolkit can be used to establish cell lines with tuneable gene expression, which, when coupled with PAT and computational modelling, can enable closed-loop control systems to deliver optimal product yield and quality.

Synthetic biology can be defined as the redesigning of existing, natural biology systems through the construction of new biological parts, devices, and systems for the purpose of improving industrial applications or biological research^4,5^. This ‘Parts, Devices, and Systems’ approach is used for systematic design, where three fundamental engineering principles are followed: (i) modularity—devices or systems are constructed from individual parts; (ii) characterisation—each part is defined in detail; and (iii) standardisation—creation of standard parts to be combined to form a device and/or system.

Two approaches can be used to apply these engineering principles in the development of biologically devices and systems: (i) top-down—the inner workings of an existing system are understood and fine-tuned; and (ii) bottom-up—a new system is created from smaller building blocks^6^. Gene expression in mammalian cells could be fine-tuned by utilising such modules, thus converting them into engineered biological machines capable of performing a range of functions. This reflects the transition in genetic engineering from focusing on individual genes to designing and manipulating entire genetic circuit systems.

As the field matures, synthetic biology has the potential to push biological boundaries by enhancing biomanufacturing capabilities, particularly in the creation of advanced cell lines^7,8^, and produce novel therapeutics. Synthetic biology provides engineers with the means to deploy circuits to easily and precisely tune the expression of multiple genes to, for example, (i) control critical quality attributes of therapeutics (such as mAbs), (ii) eliminate potential adverse off-target side effects in CAR-T cell therapies with the inclusion of safety “kill switches”^9^, and (iii) potentially solve bottleneck issues with plasmid transfection efficiencies of Adeno-Associated Virus (AAV) vectors in viral vector manufacturing.

Inducible gene expression systems are triggered by defined stimuli, while constitutive gene expression systems are continuously active. Inducible gene expression systems are favoured because they are typically reversible, thereby allowing more flexibility and conditional control. In addition, constitutive expression systems exhibit lower efficiency (e.g., leaky expression and lack of specificity) with more side effects, including cell death and delayed growth, compared to inducible gene expression systems^10^. An ideal inducible gene expression system should: (i) be orthogonal (i.e., two or more systems or components that are similar in composition and/or function do not interact with or influence one another) and specific; (ii) be inducible and reversible; (iii) have a non-toxic and bioavailable inducer; and (iv) be tuneable with low levels of noise^9^.

Designing an inducible gene expression system is challenging, and current available technology broadly categorises these systems into two groups: (i) synthetic fusions between inducible ligand receptors and DNA-binding proteins; and (ii) systems based on bacterial transcription factors^9^. A wide array of inducible genetic circuit components, including custom promoters, fusion proteins, and synthetic RNA devices, can be utilised to control cellular gene expression. Analogous to the systematic design of electronic circuits, these engineered regulatory tools have allowed for the reprogramming of cells to execute Boolean logic operations or to simulate circadian rhythm oscillations. This Review is structured to first introduce the fundamental design principles of synthetic biology (e.g., feedback, feedforward, modularity) and show how they underpin characteristic circuit behaviours such as switches, oscillators, and communication networks. Synthetic gene circuits are then classified according to their input modules (ligand-, light-, or signal-responsive) and regulatory modules (natural transcription factors, engineered DNA-binding proteins, RNA regulators), highlighting their suitability for inducible control. Computational modelling is considered a standalone toolset for prediction and design, while feedback and feedforward control are revisited as circuit-level strategies for achieving robust behaviour. Finally, applications in biopharmaceutical development and manufacturing, from cell line engineering and glycosylation control to advanced gene and cell therapies, will be discussed.

## Principles and phenomena of synthetic gene circuits

Synthetic gene circuits can be described both in terms of the design principles that underlie their operation and the behaviours (phenomena) that emerge from these principles. To avoid conflating these two levels of description, principles such as feedback, feedforward, and intercellular communication, which define how genetic components interact, will be introduced first. Then, circuit-level behaviours such as switching, oscillations, and signal processing, based on these principles, will be discussed. For example, positive feedback can generate bistable switches, negative feedback can stabilise outputs or produce oscillatory dynamics, and feedforward motifs enable noise filtering or adaptation^6^. Similarly, while switches, oscillators, and edge detectors act at the single-cell level, communication networks extend control to the population scale. This principles-to-phenomena framework links the mechanistic basis of circuit design directly to the emergent behaviours that matter for biopharmaceutical applications.

## Principles of circuit design

Before considering specific circuit behaviours such as switches or oscillators, it is useful to outline the core design principles that underpin them. Feedback, feedforward, and intercellular communication represent the fundamental motifs from which more complex regulatory behaviours emerge, and understanding these principles provides a framework for rational circuit design in mammalian systems.

Feedback is a fundamental design principle in synthetic biology, where the output of a system modifies, controls, or affects the subsequent behaviour of the same system and can be categorised into two types: positive feedback or negative feedback. Autoregulated feedback loops are self-regulating mechanisms where the system’s output directly affects its own activity or expression. In transcription, positive feedback or positive autoregulation is where a product of gene expression activates expression of the same gene^11^. Conversely, negative feedback is when a transcription factor represses its own transcription. Interestingly, the negative autoregulation motif has been extensively observed in natural transcriptional networks (~40% of known *E. coli* transcription factors incorporate autoregulation), which suggests that negative autoregulation confers evolutionary advantages^11,12^. Positive feedback has been identified as a crucial regulatory mechanism in bacterial quorum-sensing systems, allowing cells to make binary decisions in response to environmental signals^13^. Similarly, positive feedback loops have also been found in gene networks that regulate stem cell differentiation and development in eukaryotes^14–16^.

In a study comparing positively and negatively autoregulated inducible operons with unregulated systems, it was found that autoregulated systems outperformed non-regulated ones, with negative-feedback being superior to all^17^. Reduction in extrinsic transcriptional gene expression noise in negative autoregulated systems has also been experimentally demonstrated in several studies. One study used a regulatory cascade with a TetR-EGFP (enhanced green fluorescent protein) protein fusion under the control of a promoter with two tetO sites^18^. There are two circuits in this regulatory cascade: (i) the regulator circuit, which expresses TetR and represses its own expression; and (ii) the reporter circuit, which expresses the reporter protein (EGFP) when unrepressed by TetR. In the presence of an inducer, transcription of both genes is enabled because TetR does not bind to tetO^19^. In this work, it was found that noise was significantly reduced across a wide range of inducer concentrations in negative regulation^18^. Comparable results for gene circuits with a similar configuration^18^ were achieved in eukaryotic systems, including yeast (*Saccharomyces cerevisiae*) and mammalian cells^19–21^. In addition to examining noise reduction in mammalian cells, Nevozhay et al.^20^ examined the dose-response of negative feedback transcription circuits. Results from the study indicate that the relationship between protein expression and inducer concentration is governed by negative autoregulation that achieves a linear response to inducer molecule concentration^19–21^.

Gene expression timing can be regulated with cascades of feedback loops. A relatively simple cascade that consisted of the pristinamycin-responsive repressor Pip-KRAB under control of the transactivating fusion protein (tTA) was used to regulate reporter gene (SEAP) expression^22^. The pristinamycin I inducer removed the transcriptional repressor from the reporter gene promoter, while tetracycline addition suppressed Pip-KRAB expression. However, SEAP expression remained repressed by the residual Pip-KRAB, causing a time delay that could be adjusted by modulating Pip-KRAB stability. Beyond the direct implementation of feedback loops for inducible transcriptional control, feedback loops have also been used as building blocks for other types of devices and systems, such as oscillators^23–25^.

In addition to feedback control, incoherent feedforward loops (iFFLs) have been extensively implemented in synthetic biology. Unlike feedback, where inputs are adjusted based on discrepancies between the output and the desired target, feedforward control sets the input in advance, based on an understanding of how stimuli influence the process^26^. Typically, feedforward controllers comprise a sensor that detects the stimulus and an actuator that applies a predefined action in response. This architecture often connects an upstream input directly to both a target gene and an intermediate regulator that also modulates the same target. Such motifs enable adaptation, allowing cells to mount a transient response before returning to baseline even when the stimulus persists. They are also effective at filtering noise, sharpening the distinction between genuine signals and stochastic fluctuations^27^. In mammalian systems, feedforward designs have been explored to enhance the robustness of inducible circuits and reduce crosstalk, thereby supporting reliable operation under variable manufacturing conditions^28^. More detailed examples of feedforward control, including modelling frameworks and implementation strategies, will be discussed in the Modelling Frameworks Section.

Synthetic circuits are not restricted to single-cell behaviours and can be extended to populations through cell–cell communication modules. Borrowing concepts from bacterial quorum sensing or mammalian paracrine signalling, communication networks allow distributed circuits to coordinate gene expression across a culture^13^. These systems can generate collective behaviours such as synchronised oscillations, division of labour, or density-dependent induction. In the context of biopharmaceutical production, communication-based designs may help regulate population-level phenotypes, balance metabolic load, or synchronise induction across large-scale cultures^22^.

## Circuit behaviour (phenomena)

The fundamental design principles described above manifest as distinct dynamic behaviours when implemented in mammalian cells. These behaviours represent higher-order functions that emerge from the interplay of simple regulatory motifs, and they form the foundation for building more sophisticated synthetic gene circuits. By categorising behaviours according to their mechanisms (switches, Oscillators, Logic gates and edge detectors, and cell-cell communication networks), the design of genetic parts to the functional outputs required for biopharmaceutical applications can be seen.

Genetic switches or regulatory switches are a collection of genes that act to switch the states of genetic expression. The most basic type of genetic switch is a toggle switch, which enables alteration between two stable states (ON/OFF) using transient chemical inducers or thermal induction^29^. The simple switch would constitute two repressors and two inducible constitutive promoters, where each promoter is suppressed by the repressor that is produced by the opposing promoter. The bistability of toggle switches arises from the mutually inhibitory arrangement of transcriptional activation of two repressor proteins and was first demonstrated in *Escherichia coli* (*E. coli*) cells^30^.

Toggle switches have been adapted for eukaryotic cells using various expression control systems, including RNA/aptamer-based and transcription factor-based control^31,32^. The first genetic toggle switch for mammalian cells was developed using two transcriptional silencers: (i) the erythromycin inhibited Krüppel associated box (E-KRAB) domain; and (ii) the pristinamycin inhibited KRAB domain (Pip-KRAB), which responds to macrolides and streptogramins, respectively. Pip-KRAB was controlled by an E-KRAB-dependent promoter, while E-KRAB transcription was regulated by Pip-KRAB through a Pip operator site placed after the promoter for E-KRAB expression. This setup allowed only one construct to be preferentially transcribed while suppressing the other. The addition of erythromycin causes the transsilencer to unbind from its operator site, thereby activating expression of Pip-KRAB and the reporter gene, secreted alkaline phosphate (SEAP). Replacing erythromycin with streptogramin reversed transcription states through the induction of E-KRAB and inhibition of Pip-KRAB expression. Removal of any ligand did not affect the toggle switch state^32^.

Toggle switches form a synthetic memory unit—a simplified, highly controllable gene circuit for regulating cellular functions. The practicality of toggle switches lies in their transient induction (in contrast to sustained induction), which has potential applications in biopharmaceutical production and regulating critical quality attributes (CQAs), such as modulation of glycosylation profile of biotherapeutics, as well as spatiotemporal control of cell and gene therapy products^33,34^. The use of genetic switches in therapeutic cells for clinical applications has been comprehensively reviewed by Teixeira and Fussenegger in a recent series of publications^35–37^.

Oscillators cyclically alternate between two distinct states in a self-regulated and repetitive manner. While oscillation is commonly observed in electronics and physics, less examples—albeit important ones—abound in living systems. Circadian clocks in mammals are the epitome of autonomous and self-sustaining oscillatory circuits present in nature. These clocks regulate the periodic activation of target genes, enabling the 24 h rhythmic expression of gene clusters that manage day-night cycles^23,24,38^. Genetic oscillators have garnered attention due to their dynamic behaviour and their influence on metabolic pathways. Synthetic biologists have mimicked the mechanisms of endogenous oscillator circuits by placing genes under the control of specific transcription factors, generating self-sustaining oscillating expression through the interaction of transcriptional activators and repressors^39^.

In these studies, the tTA transcriptional activator was used to control a destabilised GFP variant, which provided advanced temporal resolution of gene expression. Since tTA regulates GFP transcription, tTA had to follow an oscillating expression pattern. To achieve this, tTA transcription was placed under autoregulation, forming a tTA-dependent feedforward loop that facilitated the transition between the OFF to ON state. In addition, a time-delayed negative feedback loop, regulated by prstinamycin I-dependent transactivator (PIT) and controlled by tTA, repressed tTA expression through tTA-targeting antisense RNA. This feedback allowed delayed repression of tTA in a self-regulating manner, enabling autonomous cycling of tTA induction and repression. Adjusting the number of gene templates introduced into the cells fine-tuned the frequency of this genetic oscillator^40,41^.

In a separate setup, GFP expression was controlled by the macrolide-dependent transactivator ET1, which contained an intronically encoded short hairpin RNA targeting tTA for inhibition. This RNA interference-dependent negative feedback loop produced a low-frequency oscillator with a 26 h period, resembling the natural circadian clock^23,42^. Alternative time-delayed negative feedback loops can also be created by delaying repressor transcription. In a circuit containing the Tet repressor (TetR) and a tetO promoter, a time delay in gene of interest (GOI) expression could be achieved by increasing the length of introns upstream of the two genes^43^. The added length increased transcription duration and was leveraged to adjust oscillation frequency^39,41,42^.

Previously constructed prokaryotic clocks^44–46^ employed only transcription control elements, leaving ambiguity on whether mammalian post-transcriptional control components could enable autonomous and self-sustained oscillations. Gene regulation based on antisense RNA clock-gene transcription proved to be integral to circadian machinery^44,47^. Tigges et al.^38^ achieved self-sustaining and tuneable oscillation in mammalian cells by integrating autoregulated positive transcription feedback with a two-step transcription cascade that generates non-coding antisense RNA, which facilitates autonomous translation control. These oscillatory dynamics provide deeper insights into the molecular mechanisms of biological clocks, potentially paving the way for therapeutic approaches for clock-related diseases such as Huntington’s and Alzheimer’s^45,48^.

**Fig. 1:**
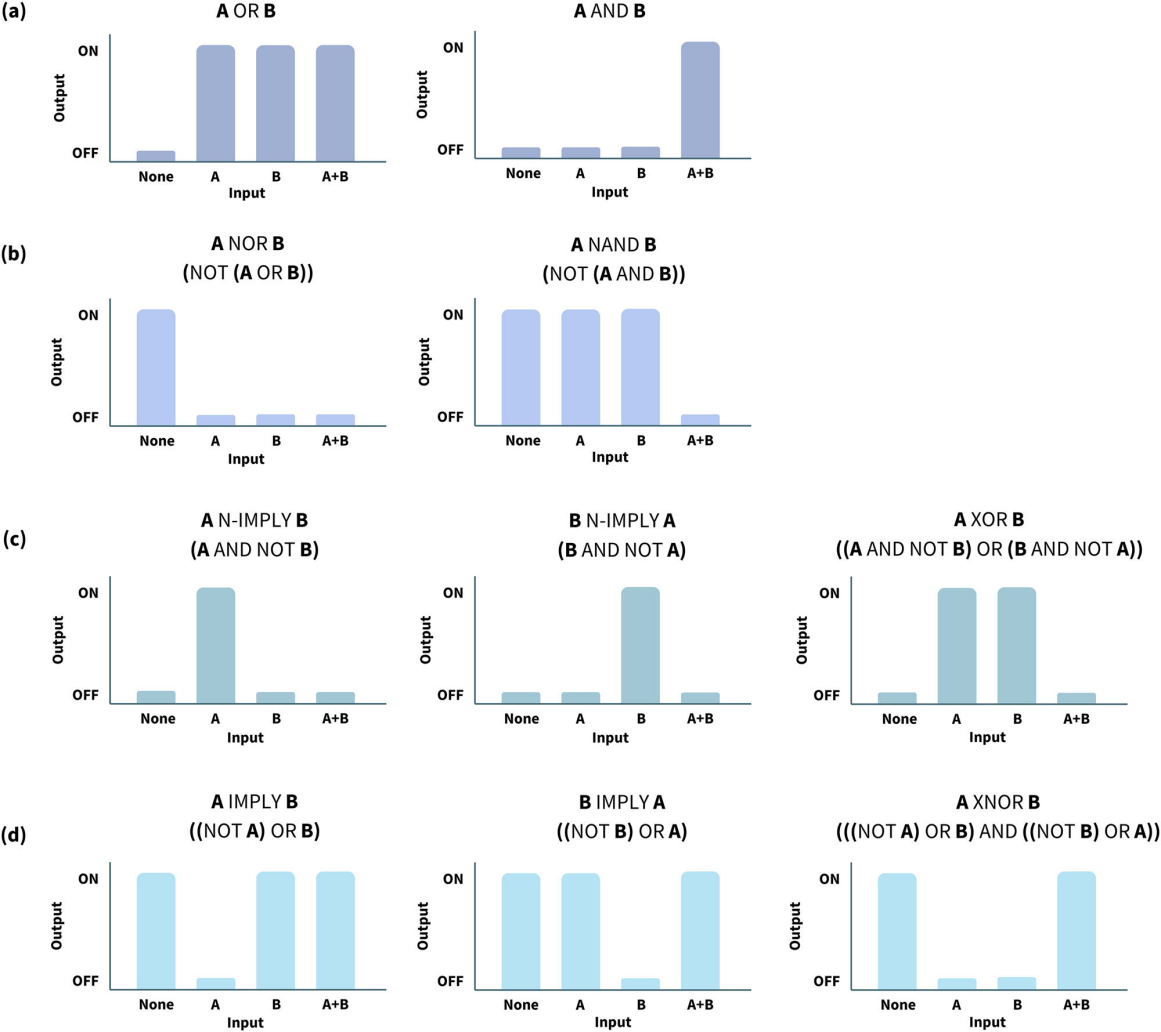
Graphical representation of OR/AND, NOR/NAND, N-IMPLY/XOR, and IMPLY/XNOR BioLogic gates, adapted from Xie et al.^163^. **a** The two fundamental gates for any computational task in electronics and biology are the two-input OR and AND gates^164,165^. OR gates permit genetic expression when either one of the input signal (A or B) is present (e.g., multiple activator-specific promoters or different mutually orthogonal activators targeting the same gene of interest)^49^, while AND gates permit genetic expression in the presence of all input signals; **b** NOT inverters are designed to prevent gene activation by excluding specific input signals from triggering expression. Therefore, NAND (A NAND B = NOT (A AND B)) and NOR (A NOR B = NOT (A OR B)) logics can be executed by inverting the AND and OR, respectively. This is achieved by incorporating a repressor-based subsystem into an active gene expression setup^49,165^, utilising synthetic promoters with multiple binding sites for various transrepressors^166^, and by using mRNAs with several target sites^51^; **c** the N-IMPLY gate (A N-IMPLY B = A AND NOT B) can be constructed using tandem trans-regulator binding sites, by combining the AND and NOT logics, such that one trans-regulator acts as an activator, while the other functions as a repressor that suppresses the activator’s role by binding targeting the same binding site^49^. The exclusive-OR (XOR) gate (A XOR B = (A AND NOT B) OR (B AND NOT A)) is a result of combining two N-IMPLY gates to permit only one input signal to activate a particular genetic event^163^; **d** similarly, two IMPLY gates (A IMPLY B = (NOT A) OR B) can be combined to create an exclusive-NOR (XNOR) gate (A XNOR B = [(NOT A) OR B] AND [(NOT B) OR A]), which enables gene expression only when both input signals are either simultaneously present or absent^163^.

In electronics, logic gates are physical devices implemented with a Boolean function to execute a logical function producing a single output based on one or more inputs. BioLogic gates (comprising coding DNA, promoters, transcription factors, RNA polymerases, non-coding RNA, DNA binding elements, and small signalling molecules) were engineered based on the electronics concept to facilitate logical transcriptional control in mammalian cells^49^. These genetic elements interact with regulatory proteins or inducers to switch the gene ON or OFF to produce RNA or protein (depending on the level of abstraction)^50^. The synthetic biologists who were the first to design logic gates in mammalian cells used a combination of tetracycline, streptogramin, macrolide, and butyrolactone to generate the BioLogic gates: OR, AND, NAND, NOR, N-IMPLY, IMPLY, XOR, and XNOR^49^ as presented in Fig. 1. Logic gates have also been effectively implemented in mammalian cells using diverse gene control systems including RNA-based^51,52^ systems and aptazymes^53^.

Communication systems are essential for coordinating events and tasks across differing populations of cells. By utilising logic-processing circuitry and cell-to-cell communication networks, Tabor et al.^54^ was able to develop a biological edge detector. Edge detection, commonly found in artificial intelligence, is a signal processing algorithm and image recognition program. The group engineered a light sensor that could enable bacteria to differentiate between light and dark regions. In the dark, the cells produced a chemical signal that diffused into illuminated areas. The deployed logic gates allow only the light-sensing cells to produce the diffusible signal and yield a positive response. Although this technology is still only emerging, it could be used in the development of cellular therapies that respond to spatiotemporal stimuli. Additionally, while the use of multiple logic gates for designing intricate transcriptional genetic control circuits offers great potential, the practical implementation of such compound circuits is limited by the scarcity of orthogonal and modular components^55^.

Cell-cell communication in multicellular organisms is crucial for the precise regulation of coordinated higher-order macroscale biological functions^56^. Pioneering work in constructing synthetic communication ecosystems was conducted in bacteria. A notable example was demonstrated by Basu et al.^57^, where receiver bacteria transiently expressed a reporter gene (GFP) in response to a sustained signal from neighbouring sender cells. Receiver cells, containing a pulse-generator circuit with a feedforward regulatory motif, respond to the inducer, acyl-homoserine lactone (AHL), synthesised by the engineered sender cells, by temporarily activating (and subsequently repressing) GFP expression. The pulse’s peak amplitude and timing were influenced by the inducer’s concentration and accumulation rate, resulting in a specific spatiotemporal response^57^. Synthetic multicellular yeast systems based on cell-cell communication networks have also been developed, where engineered ‘sender’ yeast cells synthesised a plant hormone cytokinin, which diffused to nearby ‘receiver’ yeast cells, activating a hybrid signalling pathway^58^.

To become therapeutically significant, cell-cell communication systems must be as sophisticated and reliable as natural circuits. Bacchus et al.^59^. designed communication networks that orchestrated the behaviour of individual mammalian cells through intercellular metabolic signals. The engineered circuit consists of a synthetic sender, a processor, and a receiver all of which interact with one another in ways resembling natural intercellular feedforward communication cascades. These synthetic multicellular communication systems may pave the way for future circuit-based therapies or tissue engineering strategies. Multicellular species are formed by groups of specialised cell populations that communicate for development, environmental adaptation, and maintenance. The focus of regulation mechanisms has now shifted toward understanding and mimicking quorum-sensing-based gene expression and programmed pattern formation in more complex intercellular systems beyond individual cell populations^22^.

Circuit behaviour is the predominant driver for deployment towards specific applications in the biopharmaceutical sector and beyond. The means (architecture and underlying modules) to achieve desired behaviours take on a secondary role, which may be contingent on in-house resources, processing constraints (e.g., use of chemical inducers), and IP licensing (e.g., CRISPR/ZFN/TALEN). Table 1 presents a summary of different transcriptional circuit behaviours, their characteristics, and potential applications in the biopharmaceutical sector.

**Table 1:** Circuits, characteristics and biopharmaceutical applications

Circuit Behaviour	Characteristics	Potential biopharma applications
**Switches**	Switches between states of gene expression based on defined stimuli.	• Switches can be used to fine-tune expression of glycosyltransferases to control the glycosylation profiles of therapeutic proteins. • Controlled expression of heavy and light chain genes for optimal bispecific mAb assembly. • Controlled expression of rAAV component expression for optimal assembly and encapsidation.
**Oscillators**	Cyclically alternates between distinct states of gene expression.	• Synchronise expression in replacement gene therapies for clock-related illnesses (e.g., Huntington’s and Alzheimer’s).
**Logic gates and edge detectors**	Genes expressed according to logical operations and/or spatiotemporal stimuli.	• Logic gates: engineer gene expression in T-cells with logical operators to better target tumour cells in CAR-T therapies. • Edge detectors: program cell/tissue therapies to respond to spatiotemporal stimuli (engage/activate/differentiate when arriving at therapeutic site).
**Cell-cell communication**	Gene expression is adjusted based on interactions with other cells in the population.	• Engineer production hosts to communicate and respond to environmental stress in the bioreactor. • Programming cells for enhanced interactions in tissue engineering therapies.

## Synthetic gene circuit architecture

Synthetic gene circuits in mammalian cells integrate inputs, regulatory modules, and outputs to achieve precise and tunable control of gene expression. These circuits can be designed using diverse input signals, including small molecules, hormones, or light, and are coupled to regulatory elements such as transcription factors, engineered DNA-binding proteins, or RNA-based regulators. By modularly combining these components, circuits can implement complex behaviours, such as switches, oscillators, or logic-based responses, tailored to biopharmaceutical production. This section categorises circuits according to their input sensing and regulatory mechanisms, providing a framework to understand design choices and their impact on controllable gene expression.

## Input modules

Input modules define the signals that initiate synthetic gene circuits, converting external or internal cues into measurable intracellular responses. In mammalian systems, inputs can include small molecules, hormones, cytokines, or physical stimuli such as light. These modules determine the sensitivity, dynamic range, and temporal resolution of the circuit, and their proper selection is critical for achieving precise control over gene expression.

Receptor-based circuits detect a ligand (internal but more often external) through a natural or engineered receptor. Ligand binding then activates intracellular signalling which ultimately results in activation or repression of gene expression. The first reported use of receptor sensory domain and DNA-binding protein fusions for gene expression control in mammalian cells was in the early 1990s when the exogenous yeast Gal4 transcription factor containing a DNA binding domain (DBD) was fused to an oestrogen receptor. The system was augmented with a herpes simplex viral (HSV) protein 16 (VP16) activation domain to achieve 100-fold induction and low basal activity^60^. Another study utilised ecdysone, from drosophila, as an inducer to regulate an optimised ecdysone responsive promoter^61^. Compared to tetracycline-based inducible systems, the ecdysone-based inducible system achieved less leaky expression and higher inducibility. However, ecdysone and its analogues have been reported to cause anti-apoptotic effects and influence growth in mammalian cells^62,63^.

**Fig. 2:**
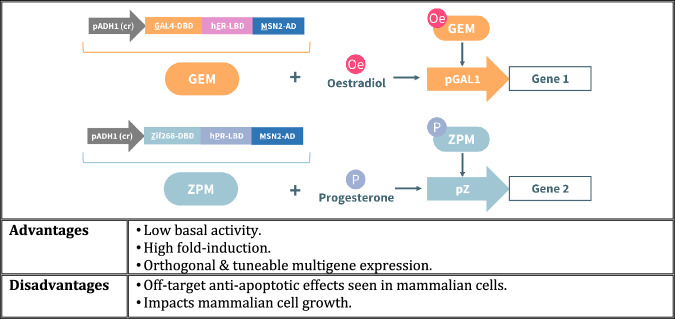
Example of a hormone-based circuit. The hormone-based system by Aranda-Díaz et al. ^64^ shows the mechanism of action of two transcriptional regulators (GEM and ZPM – a fusion of a DNA binding domain, human hormone receptor lipid binding domain, and transcriptional activation domain) in the presence of oestradiol and progesterone, respectively.

Orthogonal and tuneable multigene expression has been demonstrated in *S. cerevisiae* with synthetic hormone-inducible regulators (Fig. 2)^64^. Two separate transcriptional regulators, inducible by oestradiol and progesterone, were deployed in yeast to achieve tuneable expression of reporter proteins YFP and mKate2. Both circuits were found to act simultaneously and independently when dual-circuit cells were induced with either or both hormones. In addition, steady state protein expression was found to depend on the respective hormone concentration as opposed to having switch-like behaviour – the time taken for expression to reach steady state was dependent on the hormone-ligand binding domain, while maximum expression levels were associated with the DNA binding domain and promoter pairing. Although controlled expression of multiple genes in a tuneable, reversible, and orthogonal manner was achieved in this yeast study, human hormones produce undesirable cellular responses in mammalian cells^46,65,66^.

Optogenetic circuits utilise various wavelengths of light (a traceless inducer) to enable gene expression control. These circuits promise a higher safety and spatiotemporal precision compared to circuits that utilise chemical inducers, which can limit clinical applications due to potential side effects, issues with bioavailability, and pharmacodynamic constraints^67^. Proteins with light-sensitive domains are utilised as photosensory modules in optogenetic circuits, which can be fused to DNA binding domains (e.g., dCas9, TALENs, and transcriptional activators/repressors), to allow a response (e.g., dimerisation, oligomerisation, monomerisation, or conformational change) to light exposure^68–70^. Although recently reviewed^71,72^, this section will highlight examples of optogenetic circuits that may have applications in biopharmaceutical development and manufacture.

**Fig. 3:**
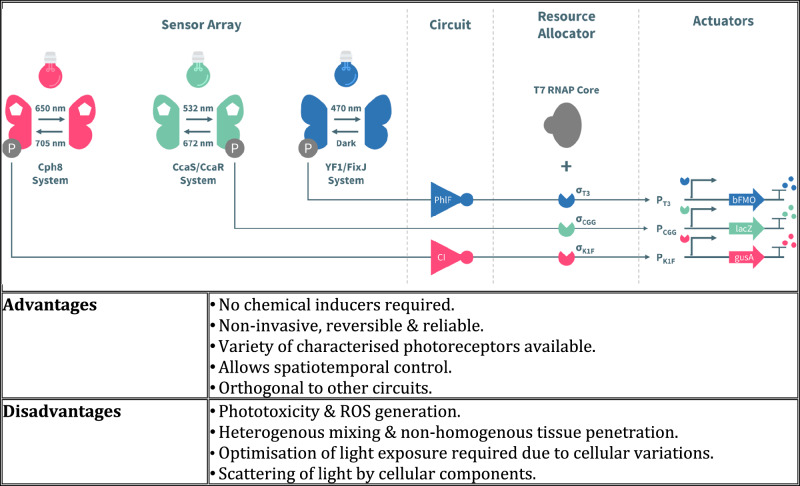
Example of an optogenetic gene expression system. A multiplexed optogenetic system is presented. It consists of four subsystems: (i) a sensor array, (ii) a circuit, (iii) a resource allocator, and (iv) actuators. Stimuli in the form of red, green, and blue light as well as darkness is transduced into signals that feed into CRISPR interference actuators that coordinate acetate production^76^.

Photoreceptors with different chromophores are essential for controlling circadian rhythm-based gene expression regulated by light sensitive opsins^73^. Rhodopsins such as channelrhodopsin-1 (ChR1) and channelrhodopsin-2 (ChR2) act as light-gated cation channels. Most natural photoreceptors use the light-oxygen-voltage (LOV) domain which is sensitive to blue light. The LOV domain is found in various light-sensitive proteins such as EL222, a 222-amino acid protein with a C-terminal helix–turn–helix (HTH) DNA-binding domain. When exposed to blue light, the LOV domain undergoes a conformational change, exposing the HTH domain to bind specific DNA sequences (C20) and initiate transcription^74^. Jayaraman et al^75^. modified the native luxI promoter by replacing the lux box with the 18-bp EL222-binding region, achieving a 5-fold transcriptional activation upon exposure to blue light. Fernandez-Rodriguez et al^76^. demonstrated a multiplexed optogenetic circuit that modulates the flux through the acetate metabolic pathway using the CRISPRi system. In this approach, a catalytically inactive dCas9 was produced in low amounts, while sgRNAs targeting the acetate production genes (pta, ackA, and poxB) were regulated by specific promoters. Cells exposed to red, green, and blue light produced less acetate than when cultured in absence of light (Fig. 3).

Optogenetics has also been used in the maturation of enzymes such as the Opto-T7RNAPs (blue light-responsive T7 RNA polymerases), which consists of photoactivatable dimerisation domains and activation in response to specific wavelength of light^77^. There are many other examples where optogenetics have been successfully deployed in prokaryotic systems to control gene expression and are not limited to the ones mentioned above. However, as the field is relatively young, limited progress has been made deploying the optogenetic toolbox in eukaryotic cells for inducible genetic control in large-scale biopharmaceutical manufacturing. This could be largely due to the challenges associated with using photoreceptors and light as an inducer.

Overexposure of light (radiant energy) at certain wavelengths can be harmful to living organisms and could lead to: (i) light toxicity; (ii) emergence of reactive oxygen species (ROS); and (iii) DNA damage and genetic instability. To circumvent these issues, photoreceptors that can be activated with short light pulses could be deployed instead^78^. However, the dose/duration of light exposure must be modulated depending on the choice of photoreceptors. A study utilising light pulsing on engineered yeast cells to activate various metabolic pathways revealed that the metabolite ratio is changed in response to the light pattern^79^. This finding demonstrates that precise tuning of the inducer can significantly impact the actuator and further support the use of the optogenetic toolbox to achieve optimal productivity in bioreactors. In addition, to avoid population heterogeneity due to reduced light penetration in high density cell cultures, the paradigm has been inverted by adapting the light-duty cycle (using light to repress metabolism)^80^. In these systems, the dark cycle alleviates repression and elicits metabolite production.

More recently, researchers have addressed the challenge of low expression levels and weak light-induced activation in mammalian cells by developing a novel blue-light-responsive transcriptional activator, DEL-VPR. This construct was created by fusing the EL222 LOV domain with three potent transcriptional activation domains: VP64, p65, and Rta. DEL-VPR enables robust light-inducible gene expression, achieving up to a 570-fold increase, which is comparable to strong constitutive promoters. It has been successfully employed to express complex monoclonal and bispecific antibodies, resulting in enhanced yield and product quality^81^. With its capacity for precise temporal control of gene expression, this system holds significant potential for both research and bioproduction applications.

Small molecules remain among the most widely used inducers in mammalian synthetic biology because of their ease of administration, tuneable dose–response, and established safety profiles in clinical contexts. Such molecules typically function by activating or deactivating engineered transcription factors, thereby linking chemical inputs to precise gene regulation. Classic examples include tetracycline/doxycycline, which enable reversible and dose-dependent control of gene expression^19,20,46^. Other molecules, such as abscisic acid, gibberellins, and synthetic ligands like cumate or pristinamycin, have expanded the repertoire of orthogonal induction systems available for mammalian use^32,82–84^. While this section introduces small molecules as the input layer, their functional implementation occurs primarily through transcription factor–based circuits, which are discussed in detail, below.

## Regulatory modules

Regulatory modules interpret signals from input modules and modulate gene expression according to a desired behaviour. These modules may use transcription factors, engineered DNA-binding proteins (e.g., zinc fingers, TALEs, CRISPR/dCas9), or RNA-based elements to control transcription, translation, or mRNA stability. By implementing feedback loops, feedforward motifs, or combinatorial logic, regulatory modules define circuit properties such as leakiness, fold-induction, response speed, and robustness. Careful design of these modules allows fine-tuning of output expression, enabling synthetic circuits to achieve precise, predictable, and scalable regulation suitable for both research and biomanufacturing applications.

Bacterial transcription factors consist of a single polypeptide linking DBDs and LBDs. Bacterial transcription factor-based systems are commonly repurposed for synthetic expression control in mammalian systems because they are simple and effective gene switches^85^, of which the tetracycline repressor (TetR) is the most widely used. Other bacterial transcription factors such as the *lac* repressor (LacI), PhlF (a TetR homologue), and cymene repressor (CymR) have been developed to complement the TetR system.

The tetracycline-controlled operator systems, ‘Tet-Off’ and ‘Tet-On’, are commonly used to regulate mammalian gene expressions. The ‘Tet-Off’ system involves the TetR protein fused to the VP16 activation domain. In absence of tetracycline (the inducer) or its analogues, the tTA transactivator binds to the operator sequence (tetO), leading to activation of downstream gene transcription. When tetracycline is present, TetR releases the operator, thus switching off gene expression. Construction of a reverse transactivator (rtTA) was conducted by isolating a TetR variant exhibiting the opposite response to tetracycline^86^. This rtTA is used in the “Tet-On” system, where gene expression occurs when the inducer is present^87^. An advantage of using non-eukaryotic transcription factors is that orthogonality can be achieved, as the specificity of transcription factor/inducer interactions is higher and crosstalk with the host mammalian cell transcriptional machinery is reduced.

A cumate-inducible system utilising CymR was developed to complement the Tet-system^83^. CymR regulates gene expression in bacteria by binding to CuO operators within the promoters of two operons, p-cym and p-cmt. A hybrid promoter designed for mammalian cells features a CuO operator placed downstream of a minimal CMV promoter. Like the TetR system, a cumate transactivator (cTA) and reverse transactivator (rcTA) were created to demonstrate modularity by fusing a VP16 activation domain to CymR and an inverse variant of CymR. The combination of CymR and rcTA resulted in a 700-fold activation in presence of cumate, and the system was used to produce high levels of therapeutic recombinant proteins, such as hCD200Fc and Rituximab, in CHO cells^84^. The cumate-inducible promoter resulted in a 4-fold higher yield of hCD200Fc compared to a constitutive promoter, thus indicating the usefulness of these tuneable inducible circuits in biopharmaceutical production^84^.

There is room for improvement in these tuneable expression systems: gene expression resembles an On/Off switch, where even small changes in inducer concentration yield large variations in protein expression^87^. Despite this, the ease of bacterial transcription factor systems is still favourable over complex receptor-based systems^88^. Systems such as lineariser circuits allow for better gene expression control by engineering the circuits to imitate the behaviour of a dimmer switch, where the addition of varying amounts of inducer yield a proportional amount of expressed protein and elicit a uniform response. A pioneering study by Nevozhay et al^20^. achieved a linear dose response using a negative feedback gene circuit in *S. cerevisiae*. The inducer used was anhydrotetracyline (ATc), which enabled transcription of the yEGFP reporter protein. Although the initial aim of the study was to establish the relationship between negative feedback regulation in transcriptional cascades and gene expression, the study used mathematical modelling predictions to show that linear dose-dependent induction can be achieved by incorporating negative autoregulation.

The *S. cerevisiae* self-repression lineariser system was then successfully adapted and deployed in the human breast adenocarcinoma MCF-7 cell line^19^. The existing framework was adapted for use in mammalian cells instead of entirely changing the circuit design, thereby displaying abstraction and modularity. A clonal population of MCF-7 cells bearing the lineariser circuit were cultured after achieving a sufficiently elevated fold-induction. A highly linear dose response (R^2^ = 0.99, L1-norm = 4.0 × 10^-2^) was obtained with a doxycycline concentration of up to 6 ng/mL. Despite comparable behaviour, the dose response was found to be linear up to 71% saturation in MCF-7 cells, in contrast to the 90% achieved in *S. cerevisiae*^20^.

**Fig. 4:**
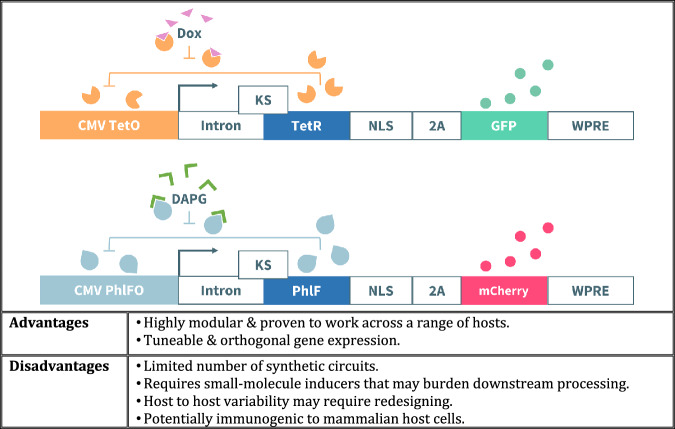
Example of a transcription factor-based circuit. Szenk et al.^46^ demonstrated the orthogonality of two negatively autoregulated lineariser gene circuits (TetR-mLin and PhlFd-mLin), where each repressor protein inhibits its own expression and the reporter protein, but addition of chemical inducers alleviates repression to yield GOI expression that responds linearly to the amount of inducer present in the system.

Linearisers based on PhlF were constructed and stably integrated into HEK293 cells to demonstrate the transferability of the TetR-based lineariser principles to other repressors^46^ (Fig. 4). The design of the PhlF linearisers was similar to the TetR linearisers in that negative autoregulation was used and both regulator and reporter (mCherry) genes are controlled by the same promoter. The PhlF lineariser achieved a: (i) maximum fold-induction of 12; (ii) linear dose response over a wider range of inducer concentrations; and (iii) low coefficient of variation (CV) of 0.38. PhlF lineariser performance was compared with the TetR lineariser expressed in HEK293 cells. The HEK293 TetR lineariser produced a steeper dose response curve, which was attributed to the higher diffusion rate of doxycycline across the cell membrane compared to the PhlF inducer (2,4-diacetylphloroglucinnol – DAPG). The TetR lineariser performance metrics were similar in HEK293 and CHO cells but had a higher fold-induction and broader linear range than in MCF-7 cells^19,46,89^, indicating the importance of characterising circuit performance across various host cells to ensure that desired outputs are achieved.

Szenk et al^46^. was able to achieve independent and orthogonal gene expression control using the TetR and PhlF linearisers. To determine orthogonality, the team: (i) tested the potential for cross-inducibility by culturing both linearisers in their opposite inducer; and (ii) determined genetic expression of both linearisers when stably integrated into the same cell and induced. It was found that reporter fluorescence was approximately equal to that of uninduced circuits in the first experiment, indicating the potential for orthogonal operation. This was established with a second experiment, as results showed that linearising ability was maintained. The success of Szenk et al^46^. could spur others to develop and expand a collection of orthogonal lineariser gene circuits to achieve simultaneous and orthogonal inducible expression of multiple genes. However, metabolic load may limit the number of deployed circuits^46^.

The above examples show that bacterial transcription factors provide orthogonal and inducible means of gene regulation in mammalian systems and, thereby, may drive advances in biopharmaceutical development and manufacturing. However, these systems raise immunogenicity concerns associated with their use of prokaryotic and viral components^46^. In addition, the use of small-molecule inducers, such as doxycycline and DAPG, may further burden downstream purification processes, and raise environmental concerns.

**Fig. 5:**
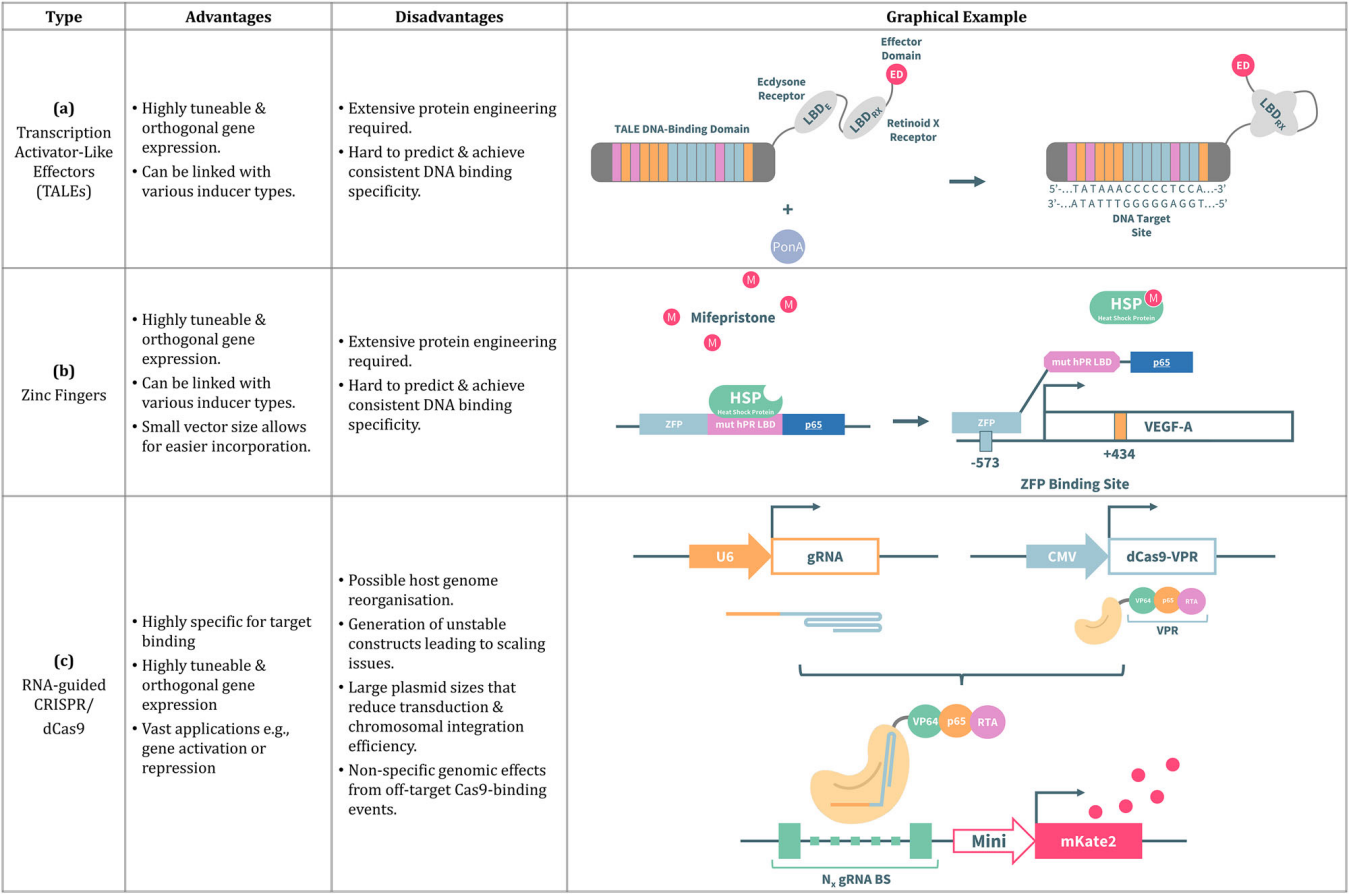
Comparison of receptor-based genetic circuits. **a** Mercer et al.^92^ fused TALE-transcription factor (TF) proteins to a chimeric single-chain retinoid X-α/ecdysone (RXE) ligand-binding domain (LBD) which, in the presence of ponasterone A (PonA), undergoes an intramolecular rearrangement and upregulates gene activation from target DNA containing a single TALE binding site; (**b**) The zinc finger protein TF remains inactive due to sequestration by heat shock proteins that only release through mifepristone binding, thus allowing zinc finger protein TF binding with the promoter^99^; (**c**) A crisprTF promoter system utilising a dCas9-VPR protein was developed to exogenously modulate mKate expression through control of gRNA transcription levels^111^.

Engineered DNA-binding proteins provide highly programmable platforms for transcriptional regulation, enabling synthetic circuits with custom specificity and modularity. Transcription activator-like effectors (TALEs) and zinc fingers (ZF) were among the earliest tools developed to target virtually any DNA sequence, paving the way for precise gene activation or repression. More recently, CRISPR/dCas9 systems have revolutionised circuit design by combining simple RNA programmability with scalable multiplexing. These DNA-binding platforms underpin many advanced regulatory modules, supporting the construction of logic gates, dynamic controllers, and multi-gene regulation strategies essential for biopharmaceutical applications. Figure 5 provides examples of these engineered-DNA-binding circuits and outlines their advantages and disadvantages.

Transcription Activator-Like Effectors (TALEs) are bacterial DNA binding proteins with significant potential for programmable gene regulation and are valued for their modular DNA recognition capabilities that allow precise binding at single-nucleotide resolution. TALEs consist of a modular DNA-binding domain (DBD), an endogenous C-terminus nuclear localisation signal sequence, and an activation domain. The DBD is typically composed of several repeats, each approximately 34 amino acid residues long^90^, and each pair of adjacent amino acids (known as repeat-variable diresidues) recognises a single nucleotide^91^. Therefore, the TALE targeting mechanism can be fused to proteins containing activation domains and ligand binding/sensory domains so that transcription can be initiated upon binding of an inducer molecule. Mercer et al^92^. achieved high tuneability with a ponasterone A (PonA) inducible system (Fig. 5a). Results from cross inducibility studies confirmed orthogonality when inducing each transcription factor with its cognate ligand. TALE transcription factors have also been designed to respond to light^93^, endogenous stimuli^94^, and small molecules^95^. However, a drawback of this technology is that extensive protein engineering is required, and large-scale application of TALEs is limited by the need to co-deliver multiple engineered factors to achieve effective transcriptional activation^96^.

Zinc finger domains are DBDs consisting of a three amino acid sequence that recognise specific nucleotides^97^. By engineering the amino acid sequence, targeted binding to any three-nucleotide sequence can be obtained^98^, and fusion of multiple zinc finger domains can target longer DNA sequences. Like the TALE-receptor system, zinc fingers can be fused with inducible regulatory domains, such as the one designed by Dent et al.^99^, where a synthetic zinc finger transcription system with mifepristone induction was developed (Fig. 5b). In this study, multigene control was achieved in HEK293 cells with zinc fingers fused with oestrogen and retinoid ecdysone receptors, and a dose-dependent response was obtained for a concentration range of different receptor configurations (50-fold to 1000-fold range of ligand concentrations). The system demonstrated independent gene expression control without cross inducibility^100^. The drawbacks of zinc finger-based systems are similar to TALEs: they are challenging to design, and consistent DNA binding specificity is difficult to predict and achieve^101^. However, zinc fingers are much smaller than TALEs, which allows for easier vector incorporation into the host cells^102^.

**Fig. 6:**
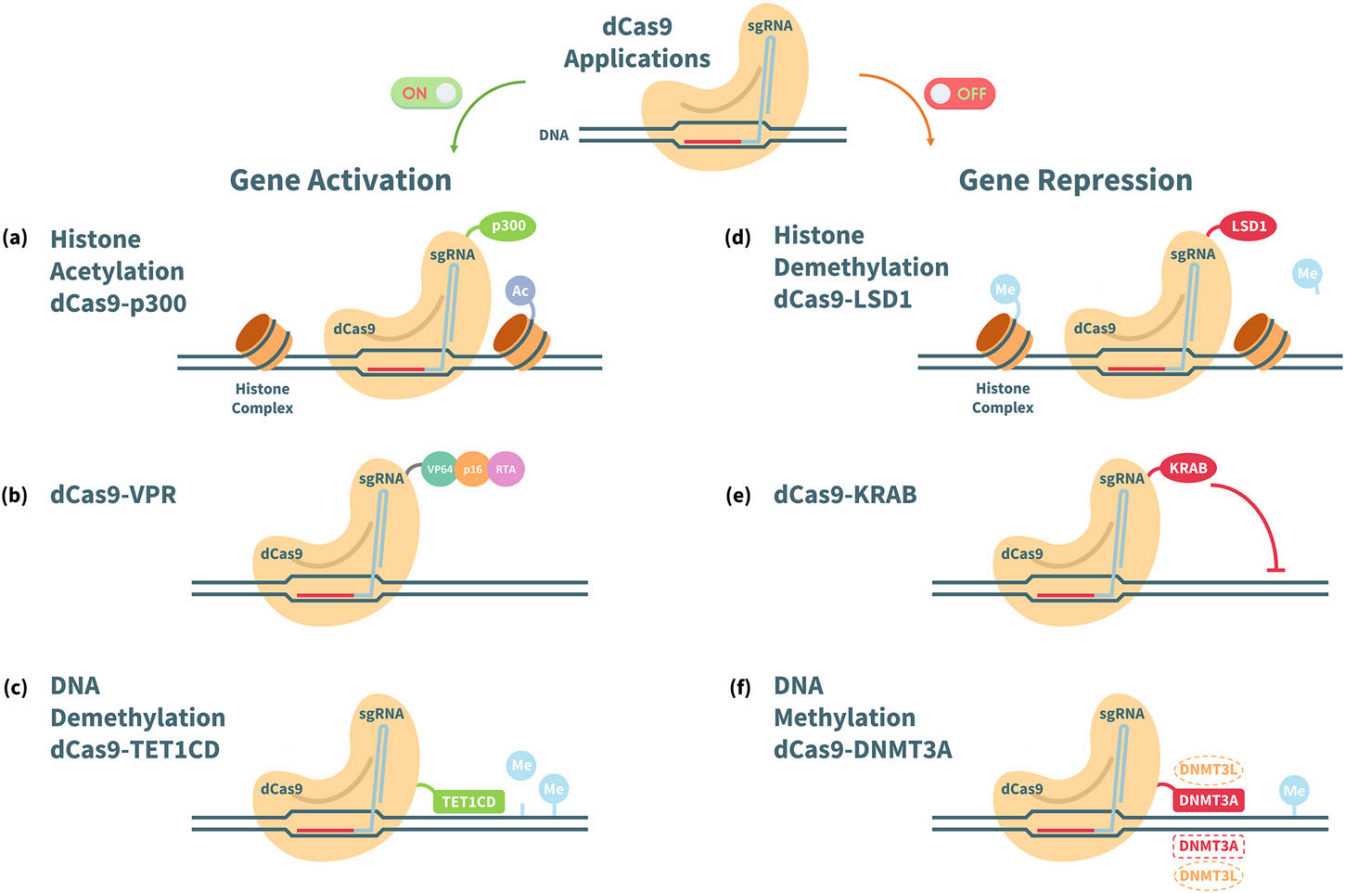
Applications of dCas9 proteins for gene activation and repression, adapted from abm Inc.^167^. **a** The dCas9 protein is combined with the catalytic core of the human E1A-associated protein p300, which acetylates histones in the genome to allow transcriptional upregulation by increasing the binding of transcription factors and RNA Polymerase II168^168^; **b** the dCas9 is linked to the tripartite activator VP64-p65-RTA, where VP64 is a synthetic tetramer derived from Herpes Simplex Viral Protein 16, p65 is a strong transcription factor, and RTA is an Epstein-Barr virus R transactivator^169^; **c** the dCas9 is fused with the catalytic domain of TET1 (Ten-eleven translocation methylcytosine dioxygenase 1) which is an enzyme that facilitates DNA demethylation, thereby allowing increased binding of transcription factors to the DNA^170^; (**d**) the dCas9 is fused to LSD1 (Lysine-specific histone demethylase) creating a gene repression system that complements the dCas9-p300 activator system^171^; (**e**) KRAB (Krüppel associated box) can be attached to dCas9 to induce transcriptional repression^107^; and **f** the dCas9 is linked to the catalytic domain of DNMT3A (DNA methyltransferase 3 alpha) that recruits a partner for dimerisation along with DNMT3L (DNA methyltransferase 3 like) proteins to methylate DNA^172^.

Programmable and targeted gene regulation has also been achieved by repurposing the CRISPR/Cas systems using ‘dead’ (catalytically inactive) Cas nucleases (RNA-guided CRISPR/dCas). The dCas9/single guide RNA (sgRNA) system can be designed to target a specified DNA sequence through nucleotide complementarity and yields minimal off-target effects^103,104^. In addition, the dCas9 proteins can be coupled to either: (i) transcription factors to activate/up-regulate gene expression; or (ii) repressors to silence genes, as depicted in Fig. 6.

Inducible CRISPRi/a (interference/activation) is a ligand-inducible controller for gene expression that was achieved by regulating the expression dCas proteins for gene targeting^105^. Gao et al^106^. achieved a dual polypeptide inducible CRISPRi/a, such that receptor dimerisation only occurs when small-molecule inducer is present by separately fusing dCas and the activation domain to single chains of either a homodimeric or heterodimeric nuclear receptor. Recovery after removal of inducer was slow and gene expression noise was not reported. It was suggested that the use of multiple activation domains could improve efficiency and reduce gene expression noise^107,108^.

Akin to the TALE and zinc finger-based systems, the CRISPR/dCas9 system aids in achieving orthogonal multigene control. Independent activation of two endogenous genes in HEK293 cells with minimal background noise was demonstrated using a chemically induced dimerising CRISPR/Cas9 system^109^. However, dose response was not measured, as the system was presented as a switch rather than a tool for fine-tuning. Kleinjan et al^110^. developed an indirectly inducible system that activated gene expression through the drug mediated degradation of dCas9. The use of different destabilising domains yielded varying performance but was able to produce a dose-dependent response and enabled multiplexed gene expression control.

A highly tuneable and comprehensive CRISPR transcription factor (crisprTF) promoter system was recently developed for the programmable regulation of gene expression in CHO cells^111^ (Fig. 5c). Two types of small molecule-inducible switches were incorporated into the RNA polymerase III promoter to exogenously modulate target gene expression through control of sgRNA transcription. The circuits demonstrated programmable episomal and chromosomal control of reporter gene expression with up to 25-fold higher activity than with the EF1α promoter^111^.

Despite the ease of multiplexed gene regulation, limitations of the inducible CRISPR/Cas system include: (i) potential changes in genome organisation from the binding of multiple sgRNAs and dCas9s to chromatin^105^; (ii) scalability issues due to generation of unstable constructs that are prone to recombination in bacteria during clonal propagation; (iii) undesired increase in plasmid sizes, and reduction in transduction and chromosomal integration efficiency due to use of multiple controls for sgRNA expression regulation^112^; and (iv) nonspecific genomic effects from off-target Cas9-binding events^113^.

RNA-based synthetic gene circuits consist of two domains: (i) an aptamer domain that detects small molecules; and (ii) an actuator domain that elicits conformational change in response to ligand binding^114,115^. The simplest device is a riboswitch – a small-molecule responsive RNA switch that regulates gene expression at the transcriptional/translational level upon inducer binding. Translation-regulating riboswitches in bacterial cells modulate access to the ribosome binding site as the two domains (aptamer and actuator) are structurally independent^116–118^. Therefore, the fundamental principles of the riboswitch could be applied to control the function of other RNA modules, such as short interfering RNAs (siRNAs) or catalytic RNA (ribozymes) in a ligand-responsive manner to drive gene silencing or even mRNA splicing.

siRNAs are short (21-23 nucleotide) RNA species that facilitates sequence-specific gene knockdown in the mammalian RNA interference (RNAi) pathway, while ribozymes can be harnessed to regulate other RNA molecules due to their effective self-cleavage activity. For example, reprogramming of siRNAs using ligand responsive ribozymes (aptamers) can be performed to construct ligand-responsive siRNA-based gene switches^119,120^. Ribozyme self-cleavage is triggered by the addition of the small-molecule ligands, such as theophylline or guanine, which causes the release of the siRNA precursors that subsequently mature into functional siRNA molecules to suppress targeted gene expression. In this way, aptamers were assembled into ribozymes to regulate the self-cleavage activity of ribozymes by ligands^121^. Rewiring of RNA inputs represents a promising strategy for designing novel gene circuits. Notably, the upregulation of certain endogenous microRNAs (miRNAs) has been linked to cancer development, making these miRNAs valuable biomarkers and therapeutic targets^122^. More recently, Klingler et al. established a library containing both natural and artificial microRNAs to fine-tune the glycosylation profile of mAbs^123^.

**Fig. 7:**
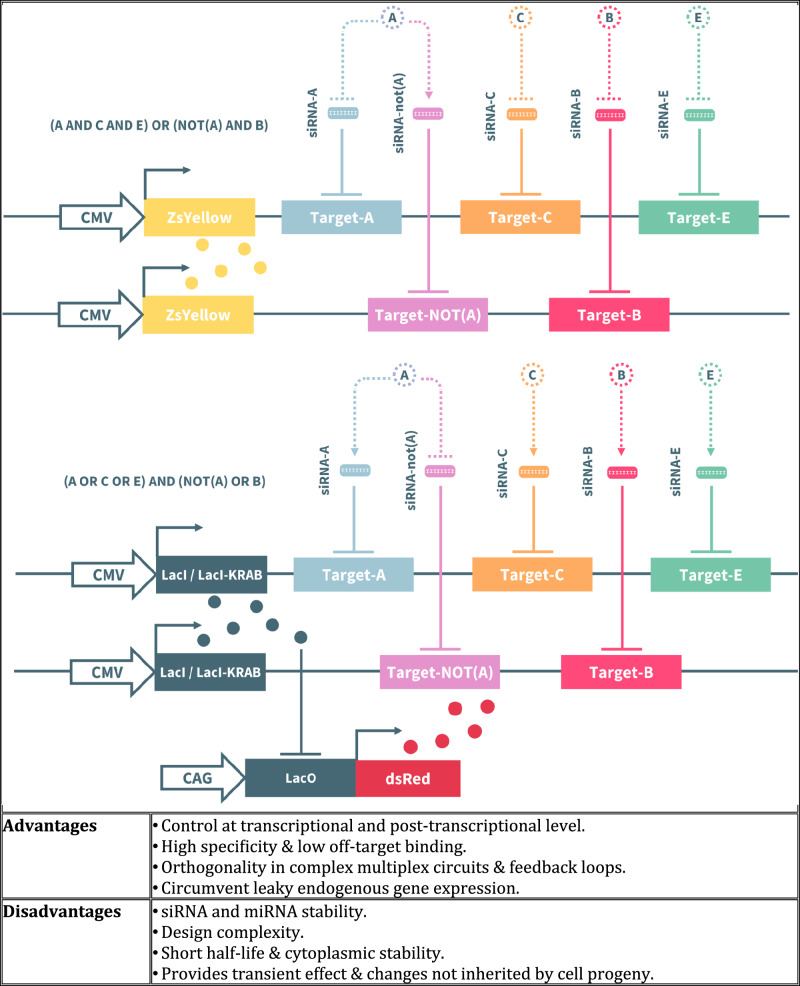
Examples of RNA-based circuits. Both circuits depict disjunctive normal form (**a**) and conjunctive normal form (**b**) expressions based on the sensory links formed between the mediator siRNAs and the various endogenous inputs present^51^.

miRNAs are especially interesting, as they are distinctly expressed in both healthy and pathological cells but cause RNAi by disrupting target mRNAs. In a novel approach that involved the creation of a circuit capable of both detecting cellular miRNA levels and responding to them, Rinaudo et al^51^. employed sequence-specific shRNA-based regulation of gene expression by incorporating miRNA-responsive elements (MREs) for oncogenic miRNAs into the 3’-untranslated region of target genes to inhibit their expression (Fig. 7). These MREs, targeting miRNAs such as miRNA-17, -21, and -30a, were placed into the noncoding regions of rtTA and LacI-Bcl2 mRNAs. As a result, elevated levels of these miRNAs led to the suppression of rtTA and LacI-Bcl2 expression and increased intracellular hBax levels to induce apoptosis^119,124,125^.

## Modelling frameworks

Mathematical modelling serves as a vital and powerful tool in synthetic biology, offering mechanistic insights into biological systems. These models allow researchers to understand, predict, and control genetic circuits. Once parameterised with experimental data, mathematical models can be used to perform in silico experiments to determine system dynamics and reduce the cost of de novo gene circuit engineering^126^. Synthetic gene circuits are generally modelled as chemical reaction systems occurring within a cell, where circuit components are produced and degraded at different rates. Deterministic and stochastic modelling strategies have proven useful in describing synthetic gene circuits^127^.

## Model-based design and optimisation of genetic circuits

Deterministic models of gene circuits are based on classical biochemical kinetics and assume that underlying quantities vary in a deterministic and continuous way^128^. These models can be represented with a set of ordinary differential equations (ODEs) which use continuous variables to describe the concentrations of RNAs, proteins, and other molecules, allowing for accurate dynamic modelling of gene regulation. There are three primary types of ODE-based modelling methods: (i) the law of mass action^129^; (ii) Hill function^130^; and (iii) Michaelis Menten Kinetics^131^. Deterministic modelling provides detailed information about system dynamics but requires experimental data for parameterisation. Such data is not always available and may be challenging to acquire^132^.

**Fig. 8:**
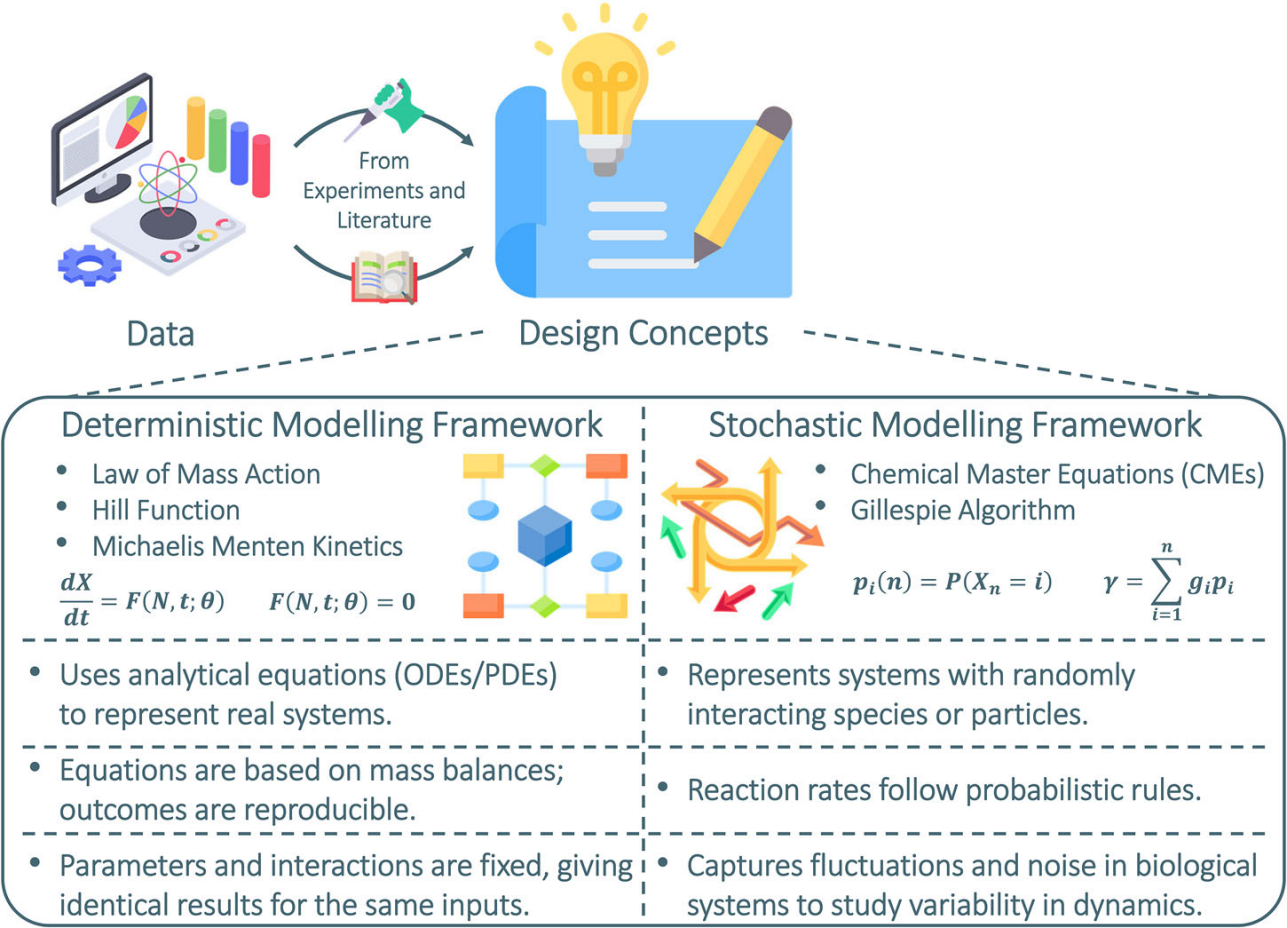
Modelling frameworks to design and optimise genetic circuits. Models can be either deterministic or stochastic, which defines the nature of the equations that underly them. Deterministic models use systems of differential algebraic equations, while stochastic models use probabilistic equations. Model development may require extensive experimental work for model validation.

A critical aspect of model-based design and optimisation of inducible gene circuits is experimental validation, as indicated at the top of Fig. 8. The extent of experimentation required for model validation will depend on circuit complexity. For example, complete model validation for a single lineariser circuit (see Transcription Factor-Based Section) would require time courses for circuit component mRNA and protein levels (repressor and GOI) as well as time courses for inducer molecule concentration. More complex systems with multiple regulatory proteins and extended crosstalk between components would require even further experimentation. However, experimental burden can be reduced through sensitivity analysis (to identify parameters that most heavily impact system states) and subsequent model-based design of optimally informative experiments^133,134^.

Given the stochastic nature of biological systems, especially in a low-number molecular context, simulations should account for inherent variability. Stochastic modelling considers the random interactions of biochemical species and uses them to capture random variations and inherent noise of the system^128^. These models use probabilistic rate laws such as Chemical Master Equations (CMEs) to describe reaction rates between species. Chemical Master Equations are usually simulated using stochastic simulation algorithms, such as the Gillepsie algorithm, which is widely used in synthetic biology^135^. Stochastic modelling is considered a more realistic approach to model gene regulatory systems, but stochastic simulations are usually computationally expensive and require information on regulatory mechanisms at the molecular level, which is not always available.

The first synthetic circuit that was designed using mathematical modelling is the toggle switch in *E. coli*^30^. The authors developed a deterministic model that captured the rate of change in repressor concentration to describe toggle switch behaviour and determine the conditions of bistability. In 2000, mathematical modelling was used to design and construct the repressilator synthetic gene circuit^136^. Here a deterministic model of six coupled first-order ODEs was used to describe the expression of three repressors (TetR, cI, LacI) and simulate the system’s temporal behaviour. The authors also employed the Gillepsie algorithm to perform stochastic simulations and investigate the effect of intrinsic noise. The model predicted that the system could exhibit oscillations across a wide parameter value range, which was in agreement with experimental data.

Stricker et al^24^. used a mathematical model to develop and robust genetic oscillator in *E. coli*. In this study, deterministic and stochastic simulations of the model indicated improved oscillations with higher doses of inducer, while the oscillation period was faster with lower inducer concentrations. The authors were able to define the parameter range over which the system would produce stable oscillations and demonstrated that time delay played a crucial role in system robustness. In particular, the parameter space where oscillatory behaviour was detected could be expanded by increasing the time delay. Both experimental and mathematical results revealed that the combination of positive and negative feedback loops promoted the robustness and tuneability of the genetic oscillator.

Mathematical modelling was also used to design and optimise the first mammalian oscillator circuit, which was based on sense-antisense positive/negative feedback regulation^38^. Here, Tigges et al. developed a deterministic mathematical model for the expression of tetracycline-controlled transactivator (tTA), pristinamycin I-dependent transactivator (PIT), and the GFP fluorescent reporter. In accordance with experiments, they predicted that the combination of positive and negative feedback loops results in sustainable and autonomous oscillations with periods of 170 min in CHO cells. In addition, the mammalian oscillator could be adjusted by modifying the doses of transfected DNA to alter frequency and amplitude. Notably, the oscillating cells exhibited significant variability in both timing and amplitude, indicating that gene expression noise impacts this dynamic process.

Nevozhay et al^20^. used a set of deterministic differential equations, based on mass-action kinetics, to develop the negative feedback lineariser circuit shown in Fig. 4. The group used their mathematical model to determine the dose-response linearisation of negative feedback transcription circuits and demonstrated that, by adding a TetR-negative autoregulation to the system, feedback can transform the dose-response curve from sigmoidal to linear. By modifying several parameters in the model (transcription rate, translation rate, degradation rate, and repressor-DNA binding rate), the authors achieved linear and precise tuning of gene expression in yeast cells. The model was later adapted to construct a mammalian version of this circuit^19^.

Feedforward controllers generally have a lower risk for instability compared with feedback controllers but tend to be less robust to input variations. Therefore, it is beneficial to have computational models that describe how stimuli affect the output. Another difference is that feedforward controllers are designed to ensure that the system’s output is stable for a specific input, but they do not account for additional stimuli that might affect the circuit. Feedforward controllers have been found to adapt to variations in template DNA abundance, thus highlighting the importance of resource loading when considering circuit transferability across different cell lines^137^.

Mathematical modelling strategies have been deployed to ‘insulate’ feedforward circuits from extraneous stimuli (e.g., variations in transcriptional and translational resource loading) and ensure their portability by making their behaviour context-independent, i.e., with robust transferability from one organism/host to another. For example, Jones et al^138^. used a deterministic modelling approach to develop an endoRNAse-based feedforward controller to adapt gene expression levels to resource loading. The resulting controller was resilient to variations in DNA copy number and achieved comparable performance when deployed in HEK293, HEK293FT, HeLa, CHO-K1, Vero 2.2, and U2OS mammalian cells.

Lillacci et al^27^. developed miRNA-based control systems which aimed to improve GOI expression robustness. The systems included (i) an incoherent feedforward circuit that allowed for tuneable output and stability against fluctuations in plasmid uptake, (ii) a negative feedback circuit that ensured GOI expression stability despite variations in transactivator dosage, and (iii) a hybrid circuit that combines the benefits of both. The authors developed an ODE-based mathematical model to analyse the different circuit topologies and compute plasmid uptake rates. Through experimental validation, the circuit achieved a 2.6-fold increase in protein expression in CHO-K1 cells and precise gene expression regulation in human induced pluripotent stem cells.

Frei et al^139^. designed and developed microRNA-based incoherent feedforward loops with enhanced gene expression stability under varying resource conditions in mammalian cells. To achieve this, this group modified an existing mathematical model^27^ to account for the effect of transcriptional and translational limitations of gene expression. Segall-Shapiro et al^140^. leveraged control-theoretic models to implement feedforward control in *E. coli* and reduce target gene variations by rejecting differences in plasmid copy numbers.

## Applications and future outlook

### Next generation cell line development

Developing novel cell lines that are tailored to production and regulatory requirements is no easy feat. Biopharmaceutical companies are constantly plagued with challenges to create and optimise their cell lines to achieve high yields of safe and efficacious products. Although the advancements in transfection, gene amplification, selection, clone isolation and testing, codon optimisation, and cell line engineering have significantly progressed over the past couple of decades, many of these strategies use screening techniques to identify cell lines that are suitable for manufacturing deployment instead of building cells that can be precisely controlled to meet manufacturing KPIs. Precise multigene control to manipulate the magnitude and timing of expression could enable the development of next-generation cell lines^7,141^. In addition, employing established vectors with multigene control will not only ensure stable expression of the desired gene, but will also streamline clone selection through inducer addition to identify high-performing clones.

### Manufacturing complex mAbs

Complex, “difficult-to-express”, protein therapeutics typically have low yields, have unpredictable manufacturability, and require engineering strategies to both product and cell line^142^. Multiple studies using co-expression of molecular chaperones, genetic effectors, and transactivators have demonstrated that product titres of these proteins could be improved. Cartwright et al^142^. determined that a combination of genetic effectors was more effective than using single genetic effectors, and optimal relative stoichiometry of these complex therapeutics is context specific. Transiently co-transfected vectors to achieve an optimal ratio of heavy to light chain expression for mAbs have demonstrated improved yields, although the optimal ratio tends to be product specific^143,144^. However, stably transfected cells are preferred, which spurred Ho et al^145^. to experiment with the internal ribosome entry site (IRES) to control translation and the heavy/light chain expression ratio. Unfortunately, this method resulted in a heterogenous product population due to high variability and poor control. Despite some unfavourable results, inducible genetic circuits are a step in the right direction to tackle issues in manufacturing complex mAbs.

### Controlling mAb glycosylation

Post translational modifications such as glycosylation are integral in the structure, function, and stability of mAbs. Therefore, the ability to control glycosylation is extremely desirable for biopharmaceutical manufacturers. Current glycoengineering efforts to control and predict glycosylation patterns lack precision and range. A promising solution to these issues is orthogonal inducible genetic circuits. Two orthogonal inducible genetic circuits were successfully used to tune fucosylation and galactosylation (from a range of 0–95% and 0–87% respectively) of an IgG1 antibody produced in CHO cells^82^. This was achieved by knocking out the endogenous glycotransferases genes (*FUT8* and β-1,4-galactosyltransferase gene, *β4GALT1*) and creating landing pads to stably integrate the synthetic inducible genetic circuits for *FUT8* and *β4GALT1*.

Compared to conventional cell line engineering strategies, inducible genetic circuits are more favourable as they do not require extensive efforts and resources to achieve the same result – development of a whole new cell line versus a single knockout and transfer of the inducible circuit into the cell. However, from a regulatory perspective, the use of these small molecule inducers may burden downstream purification. This could possibly be circumvented by incorporating a photoreceptor instead of a ligand-based one, as light (a traceless cue) is able to provide a robust, efficient, and safe way to remotely control the genetic circuits. Optogenetics present unique and novel solutions for controlling mAb glycosylation in large-scale biomanufacturing, but the technology is still emerging, and implementation may be challenging (e.g., designing and building bioreactors to homogenously distribute light throughout the vessel is a non-trivial task).

From a bioprocessing perspective, the above-described use of inducible circuits can be used for feedforward control, where the production bioreactor follows a predefined inducer or light dosing schedule to achieve a target glycosylation profile. However, this approach would require extensive circuit characterisation in bioreactor systems and, importantly, may be incapable of handling bioprocess disturbances. A more robust alternative could be feedback control, where the bioreactor is sampled periodically (e.g., every 24–48 h) to compare product glycosylation with the setpoint. The differences between the glycosylation state and the setpoint would then be used to define a control signal (e.g., inducer molecule or light dosing) to actuate the inducible circuits and control – at the gene expression level – product glycosylation. High-throughput multi-attribute methods^146,147^ could support such sampling, while the control signal would be defined by circuit characterisation (response to inducer dose), and mathematical models of cell culture and glycosylation^148,149^ could refine the control response and improve disturbance handling.

### Cell and gene therapy

There is great potential for multigene circuits to overcome the limitations of therapeutic genome editing, where regulation of temporal, contextual, and degree of gene expression are required^44^. These circuits could potentially address concerns surrounding T-cell therapies, where there could be adverse cell proliferation after transfusion and off-target effects^9^. Multigene circuits engineered into T-cells and other cell therapies could enable precise regulation of therapeutic function and provide an alternative to ‘kill switches’ for ill-behaved engineered cells^150,151^. Although expression regulation in gene therapy is a major challenge, the Rheoswitch Therapeutic System®, regulated by oral administration of Veledimex, was successfully used to achieve a localised expression of IL-12 to treat glioblastoma tumours in mice^152^. Due to the narrow therapeutic window, it was necessary to allow the regulation of gene expression. Building on this work, Monteys et al.^153^ developed a dial-like regulator for gene therapies based on RNA splicing and controlled oral administration of an inducer. Results showed a non-linear dose response in protein expression.

Apart from applications in CAR-T cell gene therapies, inducible genetic circuits could potentially overcome a major challenge in gene therapy AAV viral vector production. Standard recombinant-AAV (rAAV) production often involves calcium phosphate or polyethylenimine (PEI) transfection of: (i) two plasmids—GOI and replication/capsid (RepCap) with Helper^154^; or (ii) three plasmids—GOI, RepCap, and Helper^155^, in adherent HEK293 cells. Although these transient transfection methods are widely used, lot-to-lot variations and scalability continue to be significant challenges in rAAV production^156^.

A novel and scalable stable cell line rAAV production strategy has been developed. The method requires only one transfection with an rAAV genome plasmid that includes a drug selection marker (e.g., puromycin) and infection with an rHSV-RepCap virus. Advantages of this system include reducing production cost and duration, as only one HSV vector will be required instead of the standard two, leading to higher quality final AAV product with greater HSV clearance^157^. In addition, the empty versus full capsid ratio in the final product (a critical quality attribute of rAAVs) using this method was 50%, a marked improvement compared to transient transfection-based (16%) and 2×HSV infection-based (28%) AAV production methods^157,158^.

While stable cell line-based AAV production is promising, there is room to improve productivity. To do so, the effect of gene copy number on virus productivity was explored by transfecting cell lines with the same rAAV vector plasmid twice. Results showed no discernible difference^157^. Perhaps the plasmid containing the rAAV vector genome could be engineered into an inducible genetic circuit to increase virus productivity. By incorporating a regulatory element, the optimal inducer levels to achieve the highest virus productivity and best vector quality can be done systematically and could be aided by computational modelling^159^. In addition, inducible genetic circuits that independently regulate the expression of all components (GOI, Rep, Cap, and Helper) would allow operators to control the ratios of each expressed component to improve rAAV quality and could also reduce the duration and cost of AAV production.

A cascading, inducible gene expression system could be leveraged to optimise the production of full recombinant adeno-associated virus (rAAV) capsids by maintaining optimal gene of interest (GOI), Rep, Cap, and Helper component stoichiometry. In this configuration, expression of the component exhibiting the lowest expression rate would be activated by an external inducer (e.g., doxycycline). This, in turn, would trigger expression of subsequent components to preserve dynamic stoichiometric control. A programmable transcriptional architecture capable of achieving precise multigene expression stoichiometry has been shown to enhance the production of influenza A virus-like particles in mammalian cells^160^. Orthogonal inducible circuits could play an important role in identifying the stoichiometry that achieves optimal rAAV capsid assembly and packaging: during development, they could be used to induce different component expression levels to identify which combination achieves the highest yield of full rAAV capsids.

Within a bioprocessing framework, a stable producer cell line incorporating such a cascading inducible circuit could be expanded to high cell densities (e.g., 2 × 10⁷ cells/mL) prior to induction. rAAV production would then be initiated by inducer feeding directly to the bioreactor. This strategy would enhance the volumetric productivity of full rAAV capsids by maintaining high and consistent cell-specific productivity even under high-density culture conditions. By ensuring appropriate stoichiometry of the GOI, Rep, Cap, and Helper components, the system would support efficient rAAV assembly and packaging.

### Concluding remarks

The synthetic biology design principles of standardisation, modularity, and predictability are rapidly expanding the toolbox of components and devices to create new functionalities and adapt existing configurations to new expression systems^161^. An example would be the development of a collection of orthogonal components for mammalian cells, which have lagged behind lower eukaryotes and prokaryotes. As presented in this review, there have been substantial successes in the efforts to achieve this, e.g., the transfer of gene circuits from yeast to mammalian systems with comparable performances^19^.

However, a unified and systematic approach to component characterisation is still lacking, as there were few comparable quantitative metrics reported when evaluating gene circuit performance. Consistent reporting of standard measurements (e.g., dose response and operating context) and establishing standard protocols and precise measurement methods would further drive expansion of the parts toolbox. An example would be the adoption of a fluorescence standard to account for variations attributed to equipment type, protocols, and environmental conditions when measuring gene expression using fluorescent reporter proteins^162^.

Despite challenges associated with context dependencies, host-to-host variability, and suitability of chemical inducers, orthogonal inducible genetic circuits have vast potential in streamlining the development and manufacture of biopharmaceuticals. The application of orthogonal inducible gene circuits in the biopharmaceutical sector could revolutionise the manufacturing process, with: (i) promising solutions for the precise control of glycosylation in mAb-producing CHO cells; (ii) elimination of potentially adverse off-target side effects in CAR-T cell gene therapies; and (iii) novel methods of rAAV vector production that ensure high vector quality and virus productivity. In addition, use of computational modelling as a tool to effectively monitor, predict, and design the behaviour of the genetic circuits will undoubtedly provide manufacturers with the necessary and timely information to ensure product quality and efficacy.

## Data Availability

No datasets were generated or analysed during the current study.

## References

[1] Statista. Projected size of the biopharmaceuticals market worldwide from 2020 to 2030*. 2022; Available from: https://www.statista.com/statistics/1293077/global-biopharmaceuticals-market-size/.

[2] Walsh, G. & Walsh, E. Biopharmaceutical benchmarks 2022. *Nat. Biotechnol.* **40**, 1722–1760 (2022). 36471135 10.1038/s41587-022-01582-x PMC9735008

[3] FDA, Guidance for Industry PAT - A Framework for Innovative Pharmaceutical Development, Manufacturing, and Quality Assurance, F.A.D. Administration, Editor. 2004.

[4] Osbourn, A. E. et al. Synthetic biology. *N. Phytol.* **196**, 671–677 (2012). 10.1111/j.1469-8137.2012.04374.x 23043589

[5] Weber, W. & Fussenegger, M. Engineering of synthetic mammalian gene networks. *Chem. Biol.* **16**, 287–297 (2009). 19318210 10.1016/j.chembiol.2009.02.005

[6] Baldwin, G. et al. *Synthetic Biology—A Primer* (World Scientific Connect, 2012).

[7] Hong, J. K. et al. Towards next generation CHO cell line development and engineering by systems approaches. *Curr. Opin. Chem. Eng.* **22**, 1–10 (2018).

[8] Liu, Y. et al. Towards next-generation model microorganism chassis for biomanufacturing. *Appl. Microbiol. Biotechnol.* **104**, 1–14 (2020). 32970182 10.1007/s00253-020-10902-7

[9] Doshi, A. et al. Small-molecule inducible transcriptional control in mammalian cells. *Crit. Rev. Biotechnol.* **40**, 1131–1150 (2020). 32862714 10.1080/07388551.2020.1808583 PMC7914288

[10] Kallunki, T. et al. How to choose the right inducible gene expression system for mammalian studies? *Cells* **8**, 796 (2019). 10.3390/cells8080796 PMC6721553 31366153

[11] Goldberger, R. F. Autogenous regulation of gene expression. *Science* **183**, 810–816 (1974). 4589900 10.1126/science.183.4127.810

[12] Rosenfeld, N., Elowitz, M. B. & Alon, U. Negative autoregulation speeds the response times of transcription networks. *J. Mol. Biol.* **323**, 785–793 (2002). 12417193 10.1016/s0022-2836(02)00994-4

[13] Ferrell, J. E. Jr. et al. Simple, realistic models of complex biological processes: positive feedback and bistability in a cell fate switch and a cell cycle oscillator. *FEBS Lett.* **583**, 3999–4005 (2009). 19878681 10.1016/j.febslet.2009.10.068

[14] Osborn, D. P. et al. Cdkn1c drives muscle differentiation through a positive feedback loop with Myod. *Dev. Biol.* **350**, 464–475 (2011). 21147088 10.1016/j.ydbio.2010.12.010 PMC3044464

[15] Lee, K. E. et al. Positive feedback loop between Sox2 and Sox6 inhibits neuronal differentiation in the developing central nervous system. *Proc. Natl Acad. Sci. USA* **111**, 2794–2799 (2014). 24501124 10.1073/pnas.1308758111 PMC3932859

[16] Kueh, H. Y. et al. Positive feedback between PU.1 and the cell cycle controls myeloid differentiation. *Science* **341**, 670–673 (2013). 23868921 10.1126/science.1240831 PMC3913367

[17] Savageau, M. A. Comparison of classical and autogenous systems of regulation in inducible operons. *Nature* **252**, 546–549 (1974). 4431516 10.1038/252546a0

[18] Dublanche, Y. et al. Noise in transcription negative feedback loops: simulation and experimental analysis. *Mol. Syst. Biol.* **2**, 41 (2006). 16883354 10.1038/msb4100081 PMC1681513

[19] Nevozhay, D., Zal, T. & Balázsi, G. Transferring a synthetic gene circuit from yeast to mammalian cells. *Nat. Commun.* **4**, 1451 (2013). 23385595 10.1038/ncomms2471 PMC3573884

[20] Nevozhay, D. et al. Negative autoregulation linearizes the dose-response and suppresses the heterogeneity of gene expression. *Proc. Natl. Acad. Sci. USA* **106**, 5123–5128 (2009). 19279212 10.1073/pnas.0809901106 PMC2654390

[21] Shimoga, V. et al. Synthetic mammalian transgene negative autoregulation. *Mol. Syst. Biol.* **9**, 670 (2013). 23736683 10.1038/msb.2013.27 PMC3964311

[22] Weber, W., Kramer, B. P. & Fussenegger, M. A genetic time-delay circuitry in mammalian cells. *Biotechnol. Bioeng.* **98**, 894–902 (2007). 17461420 10.1002/bit.21463

[23] Elowitz, M. B. & Leibler, S. A synthetic oscillatory network of transcriptional regulators. *Nature* **403**, 335–338 (2000). 10659856 10.1038/35002125

[24] Stricker, J. et al. A fast, robust and tunable synthetic gene oscillator. *Nature* **456**, 516–519 (2008). 18971928 10.1038/nature07389 PMC6791529

[25] Tsai, T. Y. et al. Robust, tunable biological oscillations from interlinked positive and negative feedback loops. *Science* **321**, 126–129 (2008). 18599789 10.1126/science.1156951 PMC2728800

[26] Del Vecchio, D., Dy, A. J. & Qian, Y. Control theory meets synthetic biology. *J. R. Soc. Interface*. **13**, 20160380 (2016). 10.1098/rsif.2016.0380 PMC4971224 27440256

[27] Lillacci, G., Benenson, Y. & Khammash, M. Synthetic control systems for high performance gene expression in mammalian cells. *Nucleic Acids Res.* **46**, 9855–9863 (2018). 30203050 10.1093/nar/gky795 PMC6182142

[28] De Carluccio, G., Fusco, V. & di Bernardo, D. Engineering a synthetic gene circuit for high-performance inducible expression in mammalian systems. *Nat. Commun.* **15**, 3311 (2024). 38632224 10.1038/s41467-024-47592-y PMC11024104

[29] Perry, N. & Ninfa, A. J. Synthetic networks: oscillators and toggle switches for Escherichia coli. *Methods Mol. Biol.* **813**, 287–300 (2012). 22083749 10.1007/978-1-61779-412-4_17

[30] Gardner, T. S., Cantor, C. R. & Collins, J. J. Construction of a genetic toggle switch in Escherichia coli. *Nature* **403**, 339–342 (2000). 10659857 10.1038/35002131

[31] Auslander, S. & Fussenegger, M. Synthetic RNA-based switches for mammalian gene expression control. *Curr. Opin. Biotechnol.* **48**, 54–60 (2017). 28388465 10.1016/j.copbio.2017.03.011

[32] Kramer, B. P. et al. An engineered epigenetic transgene switch in mammalian cells. *Nat. Biotechnol.* **22**, 867–870 (2004). 15184906 10.1038/nbt980

[33] Lebar, T. et al. A bistable genetic switch based on designable DNA-binding domains. *Nat. Commun.* **5**, 5007 (2014). 25264186 10.1038/ncomms6007

[34] Kightlinger, W. et al. Synthetic glycobiology: parts, systems, and applications. *ACS Synth. Biol.* **9**, 1534–1562 (2020). 32526139 10.1021/acssynbio.0c00210 PMC7372563

[35] Galvan, S., Teixeira, A. P. & Fussenegger, M. Enhancing cell-based therapies with synthetic gene circuits responsive to molecular stimuli. *Biotechnol. Bioeng.* **121**, 2987–3000 (2024). 38867466 10.1002/bit.28770

[36] Teixeira, A. P. & Fussenegger, M. Synthetic gene circuits for regulation of next-generation cell-based therapeutics. *Adv. Sci.* **11**, e2309088 (2024). 10.1002/advs.202309088 PMC10885662 38126677

[37] Teixeira, A. P. & Fussenegger, M. Synthetic macromolecular switches for precision control of therapeutic cell functions. *Nat. Rev. Bioeng.* **2**, 1005–1022 (2024).

[38] Tigges, M. et al. A tunable synthetic mammalian oscillator. *Nature* **457**, 309–312 (2009). 19148099 10.1038/nature07616

[39] Rey, G. et al. Genome-wide and phase-specific DNA-binding rhythms of BMAL1 control circadian output functions in mouse liver. *PLoS Biol.* **9**, e1000595 (2011). 21364973 10.1371/journal.pbio.1000595 PMC3043000

[40] Ueda, H. R. et al. System-level identification of transcriptional circuits underlying mammalian circadian clocks. *Nat. Genet.* **37**, 187–192 (2005). 15665827 10.1038/ng1504

[41] Ukai-Tadenuma, M., Kasukawa, T. & Ueda, H. R. Proof-by-synthesis of the transcriptional logic of mammalian circadian clocks. *Nat. Cell Biol.* **10**, 1154–1163 (2008). 18806789 10.1038/ncb1775

[42] Swinburne, I. A. et al. Intron length increases oscillatory periods of gene expression in animal cells. *Genes Dev.* **22**, 2342–2346 (2008). 18703678 10.1101/gad.1696108 PMC2532923

[43] Purcell, O. et al. A comparative analysis of synthetic genetic oscillators. *J. R. Soc. Interface* **7**, 1503–1524 (2010). 20591848 10.1098/rsif.2010.0183 PMC2988261

[44] Krzysztoń, R. et al. Gene-circuit therapy on the horizon: synthetic biology tools for engineered therapeutics. *Acta Biochim. Pol.* **68**, 377–383 (2021). 34460209 10.18388/abp.2020_5744 PMC8590856

[45] MacDonald, I. C. & Deans, T. L. Tools and applications in synthetic biology. *Adv. Drug Deliv. Rev.* **105**, 20–34 (2016). 27568463 10.1016/j.addr.2016.08.008

[46] Szenk, M., Yim, T. & Balazsi, G. Multiplexed gene expression tuning with orthogonal synthetic gene circuits. *ACS Synth. Biol.* **9**, 930–939 (2020). 32167761 10.1021/acssynbio.9b00534 PMC7197936

[47] Weber, W. & Fussenegger, M. Inducible product gene expression technology tailored to bioprocess engineering. *Curr. Opin. Biotechnol.* **18**, 399–410 (2007). 17933507 10.1016/j.copbio.2007.09.002

[48] Jusiak, B. et al. Engineering synthetic gene circuits in living cells with CRISPR technology. *Trends Biotechnol.* **34**, 535–547 (2016). 26809780 10.1016/j.tibtech.2015.12.014

[49] Kramer, B. P., Fischer, C. & Fussenegger, M. BioLogic gates enable logical transcription control in mammalian cells. *Biotechnol. Bioeng.* **87**, 478–484 (2004). 15286985 10.1002/bit.20142

[50] Singh, V. Recent advances and opportunities in synthetic logic gates engineering in living cells. *Syst. Synth. Biol.* **8**, 271–282 (2014). 26396651 10.1007/s11693-014-9154-6 PMC4571725

[51] Rinaudo, K. et al. A universal RNAi-based logic evaluator that operates in mammalian cells. *Nat. Biotechnol.* **25**, 795–801 (2007). 17515909 10.1038/nbt1307

[52] Matsuura, S. et al. Synthetic RNA-based logic computation in mammalian cells. *Nat. Commun.* **9**, 4847 (2018). 30451868 10.1038/s41467-018-07181-2 PMC6242901

[53] Nomura, Y. & Yokobayashi, Y. Aptazyme-based riboswitches and logic gates in mammalian cells. *Methods Mol. Biol.* **2323**, 213–220 (2021). 34086283 10.1007/978-1-0716-1499-0_15

[54] Tabor, J. J. et al. A synthetic genetic edge detection program. *Cell* **137**, 1272–1281 (2009). 19563759 10.1016/j.cell.2009.04.048 PMC2775486

[55] Mills, E. M. et al. Development of mammalian cell logic gates controlled by unnatural amino acids. *Cell Rep. Methods* **1**, 100073 (2021). 35474893 10.1016/j.crmeth.2021.100073 PMC9017196

[56] Toda, S., Frankel, N. W. & Lim, W. A. Engineering cell-cell communication networks: programming multicellular behaviors. *Curr. Opin. Chem. Biol.* **52**, 31–38 (2019). 31150899 10.1016/j.cbpa.2019.04.020

[57] Basu, S. et al. Spatiotemporal control of gene expression with pulse-generating networks. *Proc. Natl Acad. Sci. USA* **101**, 6355–6360 (2004). 15096621 10.1073/pnas.0307571101 PMC404049

[58] Chen, M. T. & Weiss, R. Artificial cell-cell communication in yeast Saccharomyces cerevisiae using signaling elements from Arabidopsis thaliana. *Nat. Biotechnol.* **23**, 1551–1555 (2005). 16299520 10.1038/nbt1162

[59] Bacchus, W. et al. Synthetic two-way communication between mammalian cells. *Nat. Biotechnol.* **30**, 991–996 (2012). 22983089 10.1038/nbt.2351

[60] Braselmann, S., Graninger, P. & Busslinger, M. A selective transcriptional induction system for mammalian cells based on Gal4-estrogen receptor fusion proteins. *Proc. Natl. Acad. Sci. USA* **90**, 1657–1661 (1993). 8446579 10.1073/pnas.90.5.1657 PMC45938

[61] No, D., Yao, T. P. & Evans, R. M. Ecdysone-inducible gene expression in mammalian cells and transgenic mice. *Proc. Natl. Acad. Sci. USA* **93**, 3346–3351 (1996). 8622939 10.1073/pnas.93.8.3346 PMC39610

[62] Oehme, I., Bösser, S. & Zörnig, M. Agonists of an ecdysone-inducible mammalian expression system inhibit Fas Ligand- and TRAIL-induced apoptosis in the human colon carcinoma cell line RKO. *Cell Death Differ.* **13**, 189–201 (2006). 16082389 10.1038/sj.cdd.4401730

[63] Constantino, S. et al. The ecdysone inducible gene expression system: unexpected effects of muristerone A and ponasterone A on cytokine signaling in mammalian cells. *Eur. Cytokine Netw.* **12**, 365–367 (2001). 11399528

[64] Aranda-Díaz, A. et al. Robust synthetic circuits for two-dimensional control of gene expression in yeast. *ACS Synth. Biol.* **6**, 545–554 (2017). 27930885 10.1021/acssynbio.6b00251 PMC5507677

[65] McIsaac, R. S. et al. Synthetic gene expression perturbation systems with rapid, tunable, single-gene specificity in yeast. *Nucleic Acids Res.* **41**, e57 (2013). 23275543 10.1093/nar/gks1313 PMC3575806

[66] Ohira, M. J. et al. An estradiol-inducible promoter enables fast, graduated control of gene expression in fission yeast. *Yeast* **34**, 323–334 (2017). 28423198 10.1002/yea.3235 PMC5542879

[67] Beyer, H. M. et al. Optogenetic control of signaling in mammalian cells. *Biotechnol. J.* **10**, 273–283 (2015). 25216399 10.1002/biot.201400077

[68] Kolar, K. & Weber, W. Synthetic biological approaches to optogenetically control cell signaling. *Curr. Opin. Biotechnol.* **47**, 112–119 (2017). 28715701 10.1016/j.copbio.2017.06.010

[69] Mansouri, M., Strittmatter, T. & Fussenegger, M. Light-controlled mammalian cells and their therapeutic applications in synthetic biology. *Adv. Sci.* **6**, 1800952 (2019). 10.1002/advs.201800952 PMC6325585 30643713

[70] Rost, B. R. et al. Optogenetic tools for subcellular applications in neuroscience. *Neuron* **96**, 572–603 (2017). 29096074 10.1016/j.neuron.2017.09.047

[71] Dwijayanti, A. et al. Toward multiplexed optogenetic circuits. *Front. Bioeng. Biotechnol*. **9**, 804563 (2021). 10.3389/fbioe.2021.804563 PMC8766309 35071213

[72] Mansouri, M. & Fussenegger, M. Synthetic biology-based optogenetic approaches to control therapeutic designer cells. *Curr. Opin. Syst. Biol.* **28**, 100396 (2021).

[73] Zang, J. et al. Circadian regulation of vertebrate cone photoreceptor function. *Elife* **10**, e68903 (2021). 10.7554/eLife.68903 PMC8494479 34550876

[74] Rivera-Cancel, G., Motta-Mena, L. B. & Gardner, K. H. Identification of natural and artificial DNA substrates for light-activated LOV-HTH transcription factor EL222. *Biochemistry* **51**, 10024–10034 (2012). 23205774 10.1021/bi301306t PMC3531242

[75] Jayaraman, P. et al. Blue light-mediated transcriptional activation and repression of gene expression in bacteria. *Nucleic Acids Res.* **44**, 6994–7005 (2016). 27353329 10.1093/nar/gkw548 PMC5001607

[76] Fernandez-Rodriguez, J. et al. Engineering RGB color vision into Escherichia coli. *Nat. Chem. Biol.* **13**, 706–708 (2017). 28530708 10.1038/nchembio.2390

[77] Baumschlager, A., Aoki, S. K. & Khammash, M. Dynamic blue light-inducible T7 RNA polymerases (Opto-T7RNAPs) for precise spatiotemporal gene expression control. *ACS Synth. Biol.* **6**, 2157–2167 (2017). 29045151 10.1021/acssynbio.7b00169

[78] Lalwani, M. A. et al. Optogenetic control of the lac operon for bacterial chemical and protein production. *Nat. Chem. Biol.* **17**, 71–79 (2021). 32895498 10.1038/s41589-020-0639-1

[79] Zhao, E. M. et al. Optogenetic amplification circuits for light-induced metabolic control. *ACS Synth. Biol.* **10**, 1143–1154 (2021). 33835777 10.1021/acssynbio.0c00642 PMC8721662

[80] Zhao, E. M. et al. Optogenetic regulation of engineered cellular metabolism for microbial chemical production. *Nature* **555**, 683–687 (2018). 29562237 10.1038/nature26141 PMC5876151

[81] Gebel, J. et al. Potent optogenetic regulation of gene expression in mammalian cells for bioproduction and basic research. *Nucleic Acids Res*. **53**, gkaf546 (2025). 10.1093/nar/gkaf546 PMC12207406 40586302

[82] Chang, M. M. et al. Small-molecule control of antibody N-glycosylation in engineered mammalian cells. *Nat. Chem. Biol.* **15**, 730–736 (2019). 31110306 10.1038/s41589-019-0288-4

[83] Mullick, A. et al. The cumate gene-switch: a system for regulated expression in mammalian cells. *BMC Biotechnol.* **6**, 43 (2006). 17083727 10.1186/1472-6750-6-43 PMC1654148

[84] Poulain, A. et al. Rapid protein production from stable CHO cell pools using plasmid vector and the cumate gene-switch. *J. Biotechnol.* **255**, 16–27 (2017). 28625678 10.1016/j.jbiotec.2017.06.009

[85] Auslander, S. & Fussenegger, M. Engineering gene circuits for mammalian cell-based applications. *Cold Spring Harb Perspect Biol*. **8**, a023895 (2016). 10.1101/cshperspect.a023895 PMC4930925 27194045

[86] Gossen, M. & Bujard, H. Tight control of gene expression in mammalian cells by tetracycline-responsive promoters. *Proc. Natl. Acad. Sci. USA* **89**, 5547–5551 (1992). 1319065 10.1073/pnas.89.12.5547 PMC49329

[87] Krueger, C. et al. Tetracycline derivatives: alternative effectors for Tet transregulators. *Biotechniques* **37**, 546, 548, 550 (2004). 10.2144/04374BM04 15517964

[88] Stanton, B. C. et al. Systematic transfer of prokaryotic sensors and circuits to mammalian cells. *ACS Synth. Biol.* **3**, 880–891 (2014). 25360681 10.1021/sb5002856 PMC4277766

[89] Farquhar, K. S. et al. Role of network-mediated stochasticity in mammalian drug resistance. *Nat. Commun.* **10**, 2766 (2019). 31235692 10.1038/s41467-019-10330-w PMC6591227

[90] Mak, A. N. et al. TAL effectors: function, structure, engineering and applications. *Curr. Opin. Struct. Biol.* **23**, 93–99 (2013). 23265998 10.1016/j.sbi.2012.11.001 PMC3572262

[91] Moscou, M. J. & Bogdanove, A. J. A simple cipher governs DNA recognition by TAL effectors. *Science* **326**, 1501 (2009). 19933106 10.1126/science.1178817

[92] Mercer, A. C. et al. Regulation of endogenous human gene expression by ligand-inducible TALE transcription factors. *ACS Synth. Biol.* **3**, 723–730 (2014). 24251925 10.1021/sb400114p PMC4097969

[93] Konermann, S. et al. Optical control of mammalian endogenous transcription and epigenetic states. *Nature* **500**, 472–476 (2013). 23877069 10.1038/nature12466 PMC3856241

[94] Li, Y. et al. Transcription activator-like effector hybrids for conditional control and rewiring of chromosomal transgene expression. *Sci. Rep.* **2**, 897 (2012). 23193439 10.1038/srep00897 PMC3508452

[95] Zhao, C. et al. Multiple chemical inducible tal effectors for genome editing and transcription activation. *ACS Chem. Biol.* **13**, 609–617 (2018). 29308880 10.1021/acschembio.7b00606

[96] Black, J. B., Perez-Pinera, P. & Gersbach, C. A. Mammalian synthetic biology: engineering biological systems. *Annu Rev. Biomed. Eng.* **19**, 249–277 (2017). 28633563 10.1146/annurev-bioeng-071516-044649

[97] Malgieri, G. et al. The prokaryotic zinc-finger: structure, function and comparison with the eukaryotic counterpart. *FEBS J.* **282**, 4480–4496 (2015). 26365095 10.1111/febs.13503

[98] Martínez-Gálvez, G. et al. Deploying MMEJ using MENdel in precision gene editing applications for gene therapy and functional genomics. *Nucleic Acids Res* **49**, 67–78 (2021). 33305328 10.1093/nar/gkaa1156 PMC7797032

[99] Dent, C. L. et al. Regulation of endogenous gene expression using small molecule-controlled engineered zinc-finger protein transcription factors. *Gene Ther.* **14**, 1362–1369 (2007). 17637799 10.1038/sj.gt.3302985

[100] Magnenat, L., Schwimmer, L. J. & Barbas, C. F. 3rd, Drug-inducible and simultaneous regulation of endogenous genes by single-chain nuclear receptor-based zinc-finger transcription factor gene switches. *Gene Ther.* **15**, 1223–1232 (2008). 18528430 10.1038/gt.2008.96 PMC2705884

[101] Wolfe, S. A., Nekludova, L. & Pabo, C. O. DNA recognition by Cys2His2 zinc finger proteins. *Annu. Rev. Biophys. Biomol. Struct.* **29**, 183–212 (2000). 10940247 10.1146/annurev.biophys.29.1.183

[102] Gersbach, C. A., Gaj, T. & Barbas, C. F. Synthetic zinc finger proteins: the advent of targeted gene regulation and genome modification technologies. *Acc. Chem. Res.* **47**, 2309–2318 (2014). 24877793 10.1021/ar500039w PMC4139171

[103] Qi, L. S. et al. Repurposing CRISPR as an RNA-guided platform for sequence-specific control of gene expression. *Cell* **152**, 1173–1183 (2013). 23452860 10.1016/j.cell.2013.02.022 PMC3664290

[104] Mahas, A., Stewart, C. N. eal Jr. & Mahfouz, M. M. Harnessing CRISPR/Cas systems for programmable transcriptional and post-transcriptional regulation. *Biotechnol. Adv.* **36**, 295–310 (2018). p. 29197619 10.1016/j.biotechadv.2017.11.008

[105] Xu, X. & Qi, L. S. A CRISPR-dCas toolbox for genetic engineering and synthetic biology. *J. Mol. Biol.* **431**, 34–47 (2019). 29958882 10.1016/j.jmb.2018.06.037

[106] Gao, Y. et al. Complex transcriptional modulation with orthogonal and inducible dCas9 regulators. *Nat. Methods* **13**, 1043–1049 (2016). 27776111 10.1038/nmeth.4042 PMC5436902

[107] Gilbert, L. A. et al. CRISPR-mediated modular RNA-guided regulation of transcription in eukaryotes. *Cell* **154**, 442–451 (2013). 23849981 10.1016/j.cell.2013.06.044 PMC3770145

[108] Lu, J. et al. Multimode drug inducible CRISPR/Cas9 devices for transcriptional activation and genome editing. *Nucleic Acids Res.* **46**, e25 (2018). 29237052 10.1093/nar/gkx1222 PMC5861443

[109] Bao, Z. et al. Orthogonal genetic regulation in human cells using chemically induced CRISPR/Cas9 activators. *ACS Synth. Biol.* **6**, 686–693 (2017). 28054767 10.1021/acssynbio.6b00313

[110] Kleinjan, D. A. et al. Drug-tunable multidimensional synthetic gene control using inducible degron-tagged dCas9 effectors. *Nat. Commun.* **8**, 1191 (2017). 29084946 10.1038/s41467-017-01222-y PMC5662744

[111] Chen, W. C. W. et al. A synthetic transcription platform for programmable gene expression in mammalian cells. *Nat. Commun.* **13**, 6167 (2022). 36257931 10.1038/s41467-022-33287-9 PMC9579178

[112] Ricci, C. G. et al. Deciphering off-target effects in CRISPR-Cas9 through accelerated molecular dynamics. *ACS Cent. Sci.* **5**, 651–662 (2019). 31041385 10.1021/acscentsci.9b00020 PMC6487449

[113] Zhang, X. H. et al. Off-target Effects in CRISPR/Cas9-mediated Genome Engineering. *Mol. Ther. Nucleic Acids* **4**, e264 (2015). 26575098 10.1038/mtna.2015.37 PMC4877446

[114] Auslander, S. et al. A general design strategy for protein-responsive riboswitches in mammalian cells. *Nat. Methods* **11**, 1154–1160 (2014). 25282610 10.1038/nmeth.3136

[115] Breaker, R. R. Riboswitches: from ancient gene-control systems to modern drug targets. *Future Microbiol.* **4**, 771–773 (2009). 19722830 10.2217/fmb.09.46 PMC5340290

[116] Hanson, S. et al. Tetracycline-aptamer-mediated translational regulation in yeast. *Mol. Microbiol.* **49**, 1627–1637 (2003). 12950926 10.1046/j.1365-2958.2003.03656.x

[117] Mironov, A. S. et al. Sensing small molecules by nascent RNA: a mechanism to control transcription in bacteria. *Cell* **111**, 747–756 (2002). 12464185 10.1016/s0092-8674(02)01134-0

[118] Suess, B. et al. A theophylline responsive riboswitch based on helix slipping controls gene expression in vivo. *Nucleic Acids Res.* **32**, 1610–1614 (2004). 15004248 10.1093/nar/gkh321 PMC390306

[119] Dykstra, P. B., Kaplan, M. & Smolke, C. D. Engineering synthetic RNA devices for cell control. *Nat. Rev. Genet* **23**, 215–228 (2022). 34983970 10.1038/s41576-021-00436-7 PMC9554294

[120] Ge, H. & Marchisio, M. A. Aptamers, riboswitches, and ribozymes in *S. cerevisiae* Synthetic Biology. *Life* **11**, 248 (2021). 10.3390/life11030248 PMC8002509 33802772

[121] Wieland, M., Auslander, D. & Fussenegger, M. Engineering of ribozyme-based riboswitches for mammalian cells. *Methods* **56**, 351–357 (2012). 22305857 10.1016/j.ymeth.2012.01.005

[122] Menon, A. et al. miRNA: a promising therapeutic target in cancer. *Int. J. Mol. Sci.* 2022. **23**, 11502 (2022). 10.3390/ijms231911502 PMC9569513 36232799

[123] Klingler, F. et al. A novel system for glycosylation engineering by natural and artificial miRNAs. *Metab. Eng.* **77**, 53–63 (2023). 36906118 10.1016/j.ymben.2023.03.004

[124] Domin, G. et al. Applicability of a computational design approach for synthetic riboswitches. *Nucleic Acids Res.* **45**, 4108–4119 (2017). 27994029 10.1093/nar/gkw1267 PMC5397205

[125] Ono, H., Kawasaki, S. & Saito, H. Orthogonal protein-responsive mrna switches for mammalian synthetic biology. *ACS Synth. Biol.* **9**, 169–174 (2020). 31765565 10.1021/acssynbio.9b00343

[126] Wang, L. Z. et al. Build to understand: synthetic approaches to biology. *Integr. Biol.* **8**, 394–408 (2016). 10.1039/c5ib00252d PMC4837018 26686885

[127] Haseltine, E. L. & Arnold, F. H. Synthetic gene circuits: design with directed evolution. *Annu Rev. Biophys. Biomol. Struct.* **36**, 1–19 (2007). 17243895 10.1146/annurev.biophys.36.040306.132600

[128] MacDonald, J. T. et al. Computational design approaches and tools for synthetic biology. *Integr. Biol.* **3**, 97–108 (2011). 10.1039/c0ib00077a 21258712

[129] Koh, G. & Lee, D. Y. Mathematical modeling and sensitivity analysis of the integrated TNFalpha-mediated apoptotic pathway for identifying key regulators. *Comput. Biol. Med.* **41**, 512–528 (2011). 21632045 10.1016/j.compbiomed.2011.04.017

[130] Shao, H. et al. Systematically studying kinase inhibitor induced signaling network signatures by integrating both therapeutic and side effects. *PLoS ONE* **8**, e80832 (2013). 24339888 10.1371/journal.pone.0080832 PMC3855094

[131] Sun, X. et al. Systems modeling of anti-apoptotic pathways in prostate cancer: psychological stress triggers a synergism pattern switch in drug combination therapy. *PLoS Comput. Biol.* **9**, e1003358 (2013). 24339759 10.1371/journal.pcbi.1003358 PMC3854132

[132] Karlebach, G. & Shamir, R. Modelling and analysis of gene regulatory networks. *Nat. Rev. Mol. Cell Biol.* **9**, 770–780 (2008). 18797474 10.1038/nrm2503

[133] Franceschini, G. & Macchietto, S. Model-based design of experiments for parameter precision: state of the art. *Chem. Eng. Sci.* **63**, 4846–4872 (2008).

[134] Huang, C., Cattani, F. & Galvanin, F. An optimal experimental design strategy for improving parameter estimation in stochastic models. *Comput. Chem. Eng.* **170**, 108133 (2023).

[135] Zheng, Y. & Sriram, G. Mathematical modeling: bridging the gap between concept and realization in synthetic biology. *J. Biomed. Biotechnol.* **2010**, 541609 (2010). 20589069 10.1155/2010/541609 PMC2878679

[136] Chen, S. et al. Building robust functionality in synthetic circuits using engineered feedback regulation. *Curr. Opin. Biotechnol.* **24**, 790–796 (2013). 23566378 10.1016/j.copbio.2013.02.025 PMC3732497

[137] Bleris, L. et al. Synthetic incoherent feedforward circuits show adaptation to the amount of their genetic template. *Mol. Syst. Biol.* **7**, 519 (2011). 21811230 10.1038/msb.2011.49 PMC3202791

[138] Jones, R. D. et al. An endoribonuclease-based feedforward controller for decoupling resource-limited genetic modules in mammalian cells. *Nat. Commun.* **11**, 5690 (2020). 33173034 10.1038/s41467-020-19126-9 PMC7656454

[139] Frei, T. et al. Characterization and mitigation of gene expression burden in mammalian cells. *Nat. Commun.* **11**, 4641 (2020). 32934213 10.1038/s41467-020-18392-x PMC7492461

[140] Segall-Shapiro, T. H., Sontag, E. D. & Voigt, C. A. Engineered promoters enable constant gene expression at any copy number in bacteria. *Nat. Biotechnol.* **36**, 352–358 (2018). 29553576 10.1038/nbt.4111

[141] Brown, A. J. & James, D. C. Precision control of recombinant gene transcription for CHO cell synthetic biology. *Biotechnol. Adv.* **34**, 492–503 (2016). 26721629 10.1016/j.biotechadv.2015.12.012

[142] Cartwright, J. F. et al. A platform for context-specific genetic engineering of recombinant protein production by CHO cells. *J. Biotechnol.* **312**, 11–22 (2020). 32114154 10.1016/j.jbiotec.2020.02.012

[143] Pybus, L. P. et al. Model-directed engineering of “difficult-to-express” monoclonal antibody production by Chinese hamster ovary cells. *Biotechnol. Bioeng.* **111**, 372–385 (2014). 24081924 10.1002/bit.25116

[144] Schlatter, S. et al. On the optimal ratio of heavy to light chain genes for efficient recombinant antibody production by CHO cells. *Biotechnol. Prog.* **21**, 122–133 (2005). 15903249 10.1021/bp049780w

[145] Ho, S. C. L. et al. Control of IgG LC:HC ratio in stably transfected CHO cells and study of the impact on expression, aggregation, glycosylation and conformational stability. *J. Biotechnol.* **165**, 157–166 (2013). 23583871 10.1016/j.jbiotec.2013.03.019

[146] Carillo, S. et al. Intact multi-attribute method (iMAM): a flexible tool for the analysis of monoclonal antibodies. *Eur. J. Pharm. Biopharm.* **177**, 241–248 (2022). 35840072 10.1016/j.ejpb.2022.07.005

[147] Millan-Martin, S. et al. Comprehensive multi-attribute method workflow for biotherapeutic characterization and current good manufacturing practices testing. *Nat. Protoc.* **18**, 1056–1089 (2023). 36526726 10.1038/s41596-022-00785-5

[148] Jimenez Del Val, I., Fan, Y. & Weilguny, D. Dynamics of immature mAb glycoform secretion during CHO cell culture: an integrated modelling framework. *Biotechnol. J.* **11**, 610–623 (2016). 26743760 10.1002/biot.201400663

[149] Kotidis, P. et al. Model-based optimization of antibody galactosylation in CHO cell culture. *Biotechnol. Bioeng.* **116**, 1612–1626 (2019). 30802295 10.1002/bit.26960

[150] Re, A. Synthetic gene expression circuits for designing precision tools in oncology. *Front. Cell Dev. Biol*. **5**, 77 (2017). 10.3389/fcell.2017.00077 PMC5581392 28894736

[151] Sakemura, R. et al. A tet-on inducible system for controlling CD19-chimeric antigen receptor expression upon drug administration. *Cancer Immunol. Res.* **4**, 658–668 (2016). 27329987 10.1158/2326-6066.CIR-16-0043

[152] Barrett, J. A. et al. Regulated intratumoral expression of IL-12 using a RheoSwitch Therapeutic System(®) (RTS(®)) gene switch as gene therapy for the treatment of glioma. *Cancer Gene Ther.* **25**, 106–116 (2018). 29755109 10.1038/s41417-018-0019-0 PMC6021367

[153] Monteys, A. M. et al. Regulated control of gene therapies by drug-induced splicing. *Nature* **596**, 291–295 (2021). 34321659 10.1038/s41586-021-03770-2 PMC8966400

[154] Tang, Q. et al. Two-plasmid packaging system for recombinant adeno-associated virus. *Biores Open Access* **9**, 219–228 (2020). 33117614 10.1089/biores.2020.0031 PMC7590824

[155] Clement, N. & Grieger, J. C. Manufacturing of recombinant adeno-associated viral vectors for clinical trials. *Mol. Ther. Methods Clin. Dev.* **3**, 16002 (2016). 27014711 10.1038/mtm.2016.2 PMC4804725

[156] van der Loo, J. C. & Wright, J. F. Progress and challenges in viral vector manufacturing. *Hum. Mol. Genet.* **25**, R42–R52 (2016). 26519140 10.1093/hmg/ddv451 PMC4802372

[157] Selvaraj, N. et al. Detailed protocol for the novel and scalable viral vector upstream process for AAV gene therapy manufacturing. *Hum. Gene Ther.* **32**, 850–861 (2021). 33397196 10.1089/hum.2020.054 PMC8418526

[158] Adamson-Small, L. et al. A scalable method for the production of high-titer and high-quality adeno-associated type 9 vectors using the HSV platform. *Mol. Ther. Methods Clin. Dev.* **3**, 16031 (2016). 27222839 10.1038/mtm.2016.31 PMC4863725

[159] Nguyen, T. N. T. et al. Mechanistic model for production of recombinant adeno-associated virus via triple transfection of HEK293 cells. *Mol. Ther. Methods Clin. Dev.* **21**, 642–655 (2021). 34095346 10.1016/j.omtm.2021.04.006 PMC8143981

[160] Qin, C. et al. Precise programming of multigene expression stoichiometry in mammalian cells by a modular and programmable transcriptional system. *Nat. Commun.* **14**, 1500 (2023). 36932109 10.1038/s41467-023-37244-y PMC10023750

[161] Slusarczyk, A. L., Lin, A. & Weiss, R. Foundations for the design and implementation of synthetic genetic circuits. *Nat. Rev. Genet.* **13**, 406–420 (2012). 22596318 10.1038/nrg3227

[162] Resch-Genger, U., Hoffmann, K. & Hoffmann, A. Standardization of fluorescence measurements: criteria for the choice of suitable standards and approaches to fit-for-purpose calibration tools. *Ann. N. Y. Acad. Sci.* **1130**, 35–43 (2008). 18596329 10.1196/annals.1430.018

[163] Xie, M. & Fussenegger, M. Designing cell function: assembly of synthetic gene circuits for cell biology applications. *Nat. Rev. Mol. Cell Biol.* **19**, 507–525 (2018). 29858606 10.1038/s41580-018-0024-z

[164] Green, A. A. et al. Complex cellular logic computation using ribocomputing devices. *Nature* **548**, 117–121 (2017). 28746304 10.1038/nature23271 PMC6078203

[165] Tamsir, A., Tabor, J. J. & Voigt, C. A. Robust multicellular computing using genetically encoded NOR gates and chemical ‘wires. *Nature* **469**, 212–215 (2011). 21150903 10.1038/nature09565 PMC3904220

[166] Gaber, R. et al. Designable DNA-binding domains enable construction of logic circuits in mammalian cells. *Nat. Chem. Biol.* **10**, 203–208 (2014). 24413461 10.1038/nchembio.1433

[167] abm Inc. Gene Regulation with dCas9. https://info.abmgood.com/crispr-cas9-gene-regulation-dCas9 (2017).

[168] Zentner, G. E. & Henikoff, S. Epigenome editing made easy. *Nat. Biotechnol.* **33**, 606–607 (2015). 26057978 10.1038/nbt.3248

[169] Chavez, A. et al. Comparison of Cas9 activators in multiple species. *Nat. Methods* **13**, 563–567 (2016). 27214048 10.1038/nmeth.3871 PMC4927356

[170] Xu, X. et al. A CRISPR-based approach for targeted DNA demethylation. *Cell Discov.* **2**, 16009 (2016). 27462456 10.1038/celldisc.2016.9 PMC4853773

[171] Kearns, N. A. et al. Functional annotation of native enhancers with a Cas9-histone demethylase fusion. *Nat. Methods* **12**, 401–403 (2015). 25775043 10.1038/nmeth.3325 PMC4414811

[172] Vojta, A. et al. Repurposing the CRISPR-Cas9 system for targeted DNA methylation. *Nucleic Acids Res.* **44**, 5615–5628 (2016). 26969735 10.1093/nar/gkw159 PMC4937303

