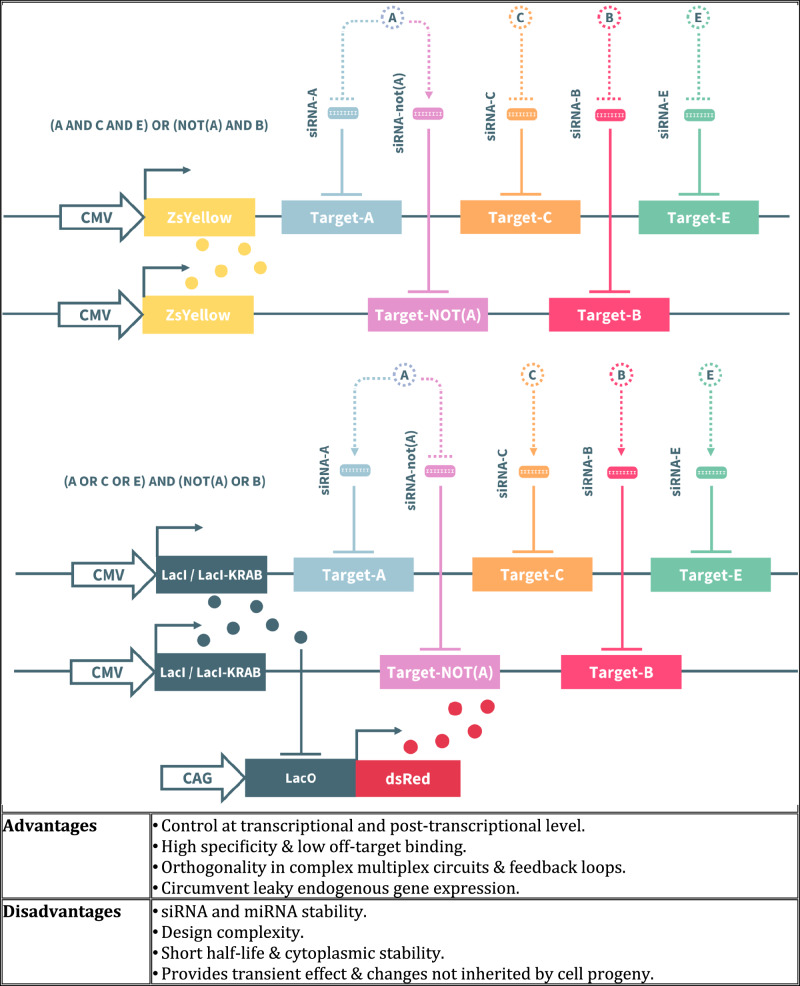# Publisher Correction: Mammalian synthetic gene circuits for biopharmaceutical development & manufacture

**DOI:** 10.1038/s41540-026-00659-6

**Published:** 2026-02-27

**Authors:** Sheryl Li Yan Lim, Sofia Gialamoidou, Rajinder Kaur, Ioscani Jimenez del Val

**Affiliations:** School of Chemical & Bioprocess Engineering, University College Dublin D04 V1W8, Dublin, Ireland

**Keywords:** Biological techniques, Biotechnology, Computational biology and bioinformatics, Engineering

Correction to: *npj Systems Biology and Applications* 10.1038/s41540-025-00621-y, published online 02 December 2025

In this article Fig(s) 2, 3, 4 and 7 appeared incorrectly and have now been corrected in the original publication. For completeness and transparency, the old incorrect versions are displayed below.

Incorrect Figure 2
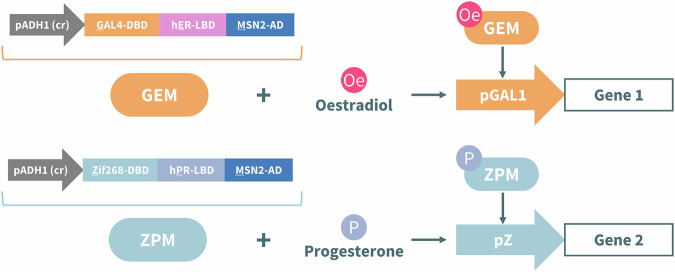


Correct Figure 2
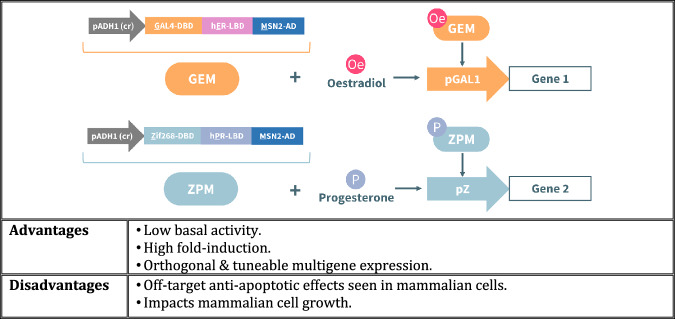


Incorrect Figure 3
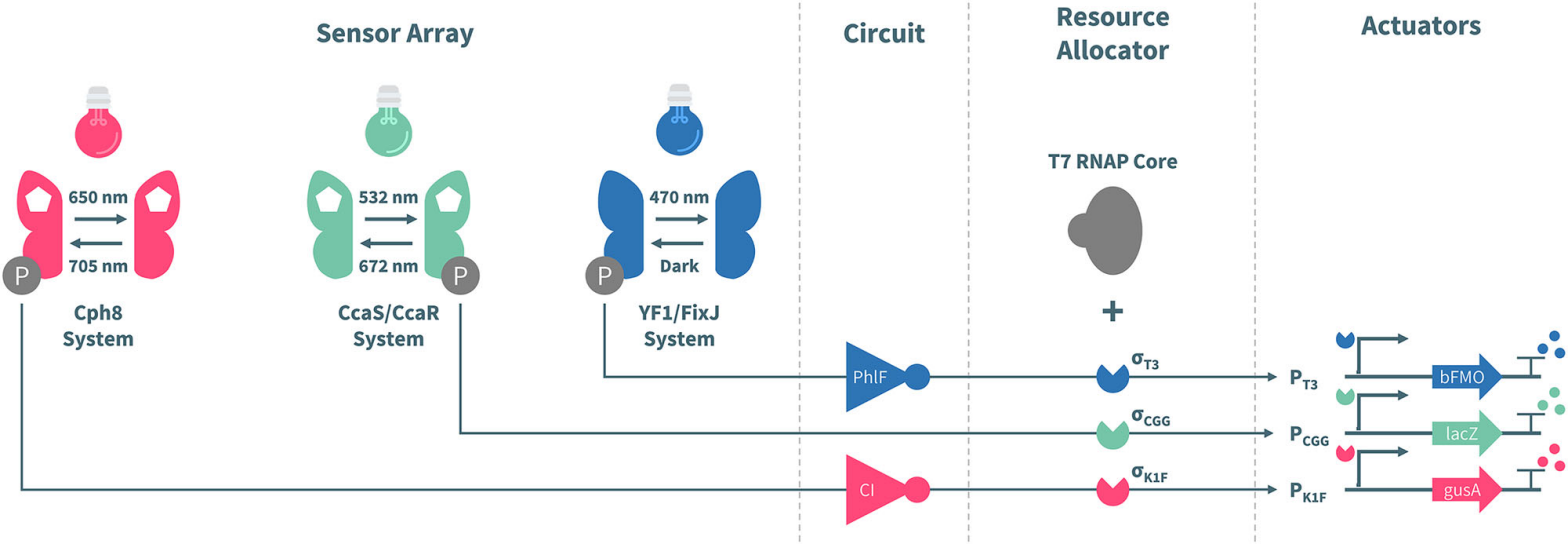


Correct Figure 3
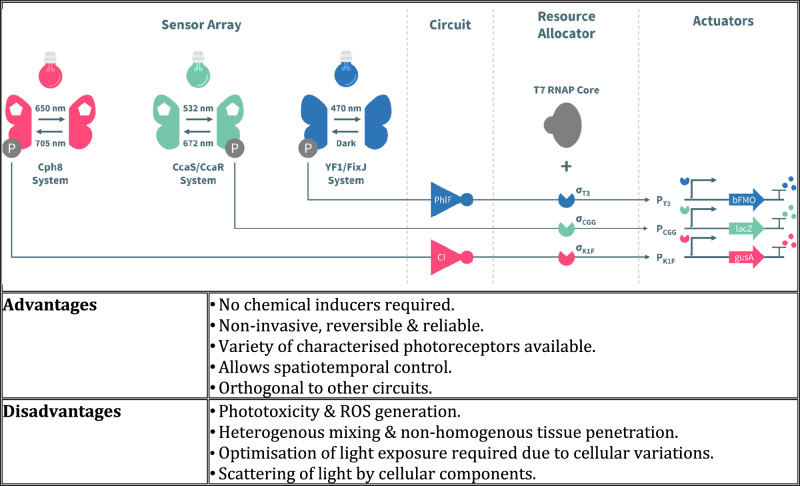


Incorrect Figure 4
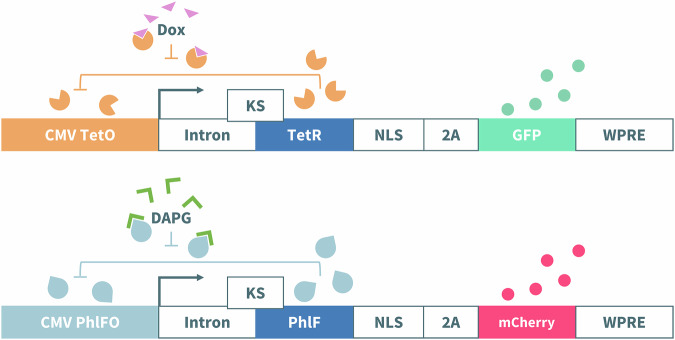


Correct Figure 4
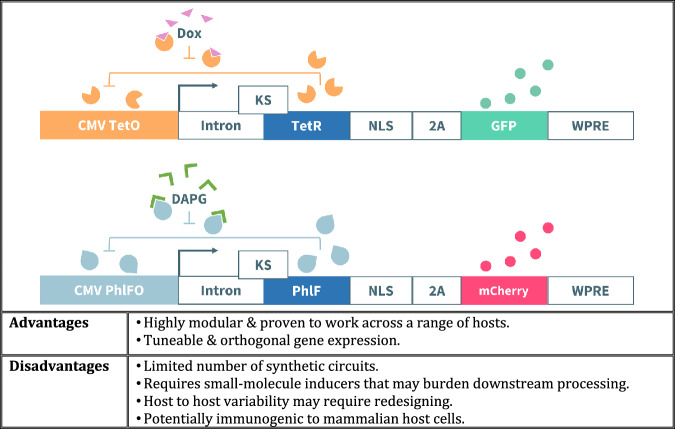


Incorrect Figure 7
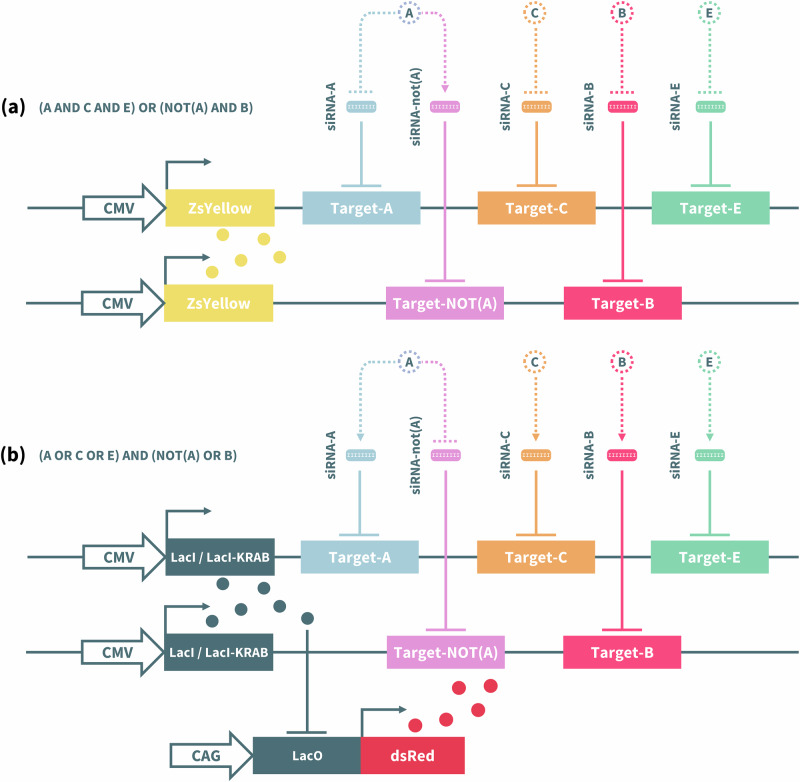


Correct Figure 7